# Septal Class A Penicillin-Binding Protein Activity and ld-Transpeptidases Mediate Selection of Colistin-Resistant Lipooligosaccharide-Deficient Acinetobacter baumannii

**DOI:** 10.1128/mBio.02185-20

**Published:** 2021-01-05

**Authors:** Katie N. Kang, Misha I. Kazi, Jacob Biboy, Joe Gray, Hannah Bovermann, Jessie Ausman, Cara C. Boutte, Waldemar Vollmer, Joseph M. Boll

**Affiliations:** a Department of Biology, University of Texas Arlington, Arlington, Texas, USA; b Centre for Bacterial Cell Biology, Biosciences Institute, Newcastle University, Newcastle upon Tyne, United Kingdom; c Biosciences Institute, Newcastle University, Newcastle upon Tyne, United Kingdom; University of Texas Southwestern Medical Center Dallas

**Keywords:** penicillin-binding protein 1A, ld-transpeptidase, peptidoglycan, lipooligosaccharide, Gram-negative, *Acinetobacter*, septation

## Abstract

Despite dogma suggesting that lipopolysaccharide/lipooligosaccharide (LOS) was essential for viability of Gram-negative bacteria, several Acinetobacter baumannii clinical isolates produced LOS^−^ colonies after colistin selection. Inactivation of the conserved class A penicillin-binding protein, PBP1A, was a compensatory mutation that supported isolation of LOS^−^ A. baumannii, but the impact of PBP1A mutation was not characterized. Here, we show that the absence of PBP1A causes septation defects and that these, together with ld-transpeptidase activity, support isolation of LOS^−^ A. baumannii. PBP1A contributes to proper cell division in A. baumannii, and its absence induced cell chaining. Only isolates producing three or more septa supported selection of colistin-resistant LOS^−^ A. baumannii. PBP1A was enriched at the midcell, where the divisome complex facilitates daughter cell formation, and its localization was dependent on glycosyltransferase activity. Transposon mutagenesis showed that genes encoding two putative ld-transpeptidases (LdtJ and LdtK) became essential in the PBP1A mutant. Both LdtJ and LdtK were required for selection of LOS^−^ A. baumannii, but each had distinct enzymatic activities in the cell. Together, these findings demonstrate that defects in PBP1A glycosyltransferase activity and ld-transpeptidase activity remodel the cell envelope to support selection of colistin-resistant LOS^−^ A. baumannii.

## INTRODUCTION

The Gram-negative cell envelope is tripartite with an inner (cytoplasmic) membrane, a periplasm that includes a thin peptidoglycan layer, and an outer membrane, which is enriched with surface-exposed lipopolysaccharide (LPS) or lipooligosaccharide (LOS). The cell envelope maintains cell shape (1, 2), supports the mechanical load caused by the turgor (3), and enables the cell to rapidly adapt to environmental challenges. Specialized macromolecular complexes span the cell envelope and coordinate peptidoglycan biosynthesis and LPS/LOS localization.

LPS/LOS is assembled at the inner membrane (4–6) and transported to the outer membrane via LptA-G, which bridges the periplasm and peptidoglycan cell wall (7–12). LPS/LOS glycolipids are based on a highly conserved lipid A moiety that anchors them on the surface-exposed face of the outer membrane. LPS/LOS disruption leads to rapid lysis and death; therefore, it has been targeted with antimicrobials (13). For example, colistin (polymyxin E) is a last-resort antimicrobial used to treat multidrug-resistant bacteria, including the ESKAPE pathogen Acinetobacter baumannii (14, 15). Colistin binds the lipid A phosphate groups to perturb the outer membrane barrier, which rapidly kills the bacterium (16, 17). Surprisingly, several A. baumannii clinical isolates inactivate LOS biosynthesis and rapidly establish resistance to otherwise toxic colistin concentrations (18, 19). However, certain A. baumannii clinical isolates could not develop such resistance. Previous work showed that inactivation of penicillin-binding protein 1A (PBP1A) (encoded by *mrcA*) is a compensatory mutation that supports selection of colistin-resistant LOS^−^ A. baumannii (19), but the role of PBP1A in A. baumannii physiology and how PBP1A mutation alters the cell envelope have not been reported.

In Escherichia coli, PBP1A and PBP1B (encoded by *mrcB*) are semi-redundant class A penicillin-binding proteins (aPBPs). aPBPs are bifunctional enzymes, catalyzing both polymerization of glycans via glycosyltransferase (GTase) activity and cross-linking of stem peptides by dd-transpeptidase (dd-TPase) activity. The dd-TPase activity of aPBPs depends on ongoing GTase reactions (20), and in E. coli aPBP activity contributes to a substantial amount of the peptidoglycan synthesized per generation (21). PBP1B not only contributes to peptidoglycan maintenance (22) but also interacts with the divisome complex (23, 24), which forms the septum and constricts the cell envelope at the midcell (23, 25). PBP1A interacts with PBP2, which suggests it has a role in cell elongation during growth (26). While PBP1A and PBP1B have distinct roles in growth, only one enzyme is required for growth because the other can compensate (26–28). Specifically, in the absence of PBP1B, PBP1A was enriched at the midcell (26), which implies PBP1A compensates to rescue cell division defects.

In E. coli, the majority of transpeptidation reactions in peptidoglycan are catalyzed by PBPs to result in 4-3 cross-links. However, ld-transpeptidases (ld-TPases) such as LdtD, LdtE, and LdtF form 3-3 cross-links (29–31). 3-3 cross-linking becomes essential for E. coli survival when LPS transport or biosynthesis is disrupted, presumably because LdtDEF repair peptidoglycan defects together with the GTase function of PBP1B and the dd-carboxypeptidase PBP6a (29). In contrast, LdtA, LdtB, and LdtC attach the outer membrane-anchored Braun’s lipoprotein (Lpp) to peptidoglycan, which stabilizes the cell envelope (32).

A. baumannii encodes two aPBPs, PBP1A and PBP1B, and two putative ld-TPases (LdtJ and LdtK), but their roles in growth and division have not been characterized. Here, we show that only multiseptated A. baumannii strains can support LOS^−^ colony formation. In contrast to E. coli (26), A. baumannii PBP1A is required for proper cell division and PBP1B is unable to compensate in its absence, which enables PBP1A mutants to assemble multiple septal sites. Specifically, disruption of PBP1A GTase activity produces the septation defect, which is also characteristic of LOS^−^ A. baumannii. PBP1A-mCherry and PBP1A_S459A_-mCherry (dd-TPase mutant) localize at the midcell during growth, where PBP1A potentially interacts with divisome components to synthesize septal peptidoglycan during division. In contrast, PBP1A_E92Q_-mCherry (GTase mutant) did not localize at the midcell, suggesting GTase activity is required for septal localization. In addition to a role in cell division, the PBP1A mutant was resistant to several β-lactam antibiotics relative to wild type and the PBP1B mutant, indicating PBP1A contributes to intrinsic β-lactam susceptibility. Furthermore, *ldtJ* and *ldtK* gene deletions were synthetically lethal in the Δ*mrcA* mutant and were essential for selection of colistin-resistant LOS^−^ A. baumannii. LdtJ forms 3-3 cross-links and incorporates d-amino acids onto peptidoglycan stem peptides, while LdtK stabilizes the outer membrane. Together, slowed septation, alternative cross-linking, and outer membrane stabilization support colistin selection of LOS^−^ A. baumannii.

## RESULTS

### Isolation of colistin-resistant LOS^**−**^ A. baumannii correlates with defective septation.

**FIG 1:**
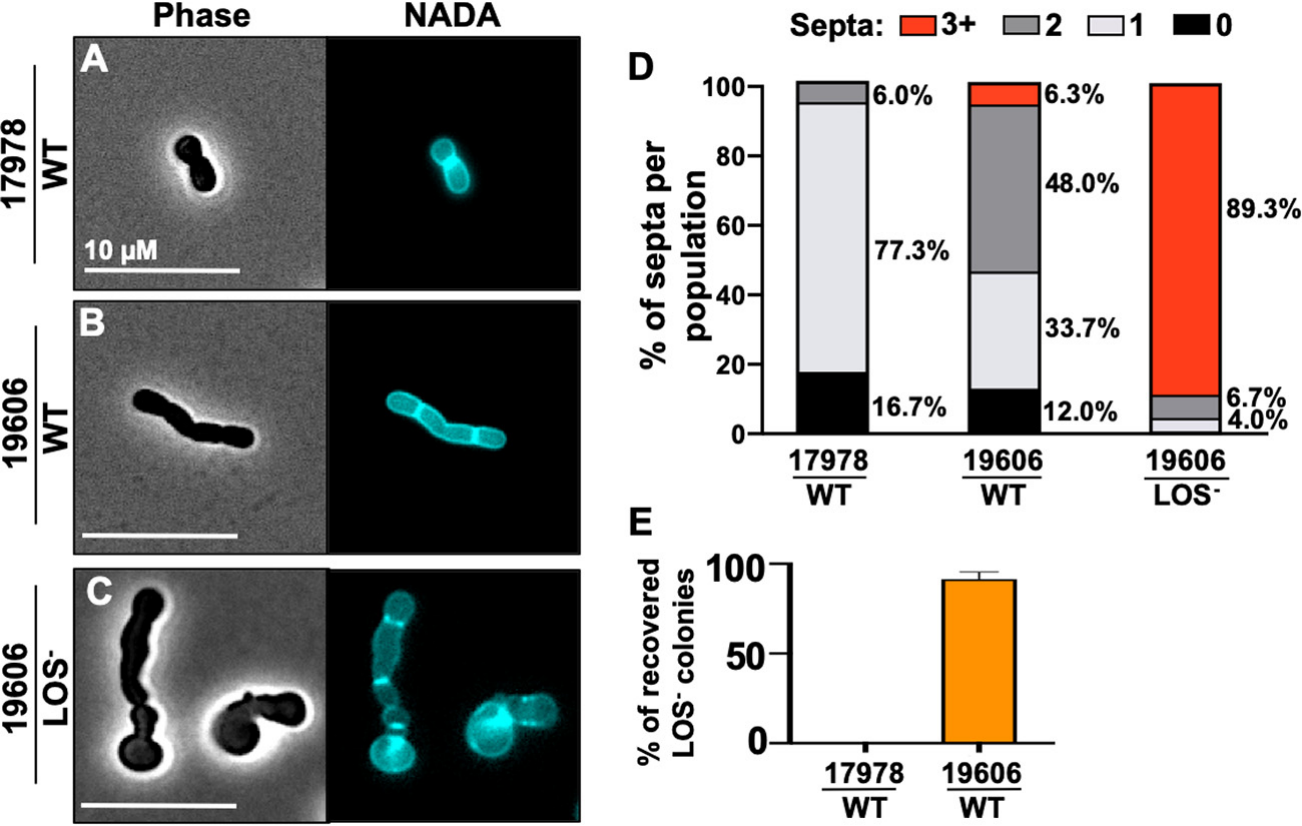
Microscopy of A. baumannii 17978 and 19606 in logarithmic growth phase. (A to C) Phase and fluorescence microscopy of wild-type (WT) 17978 (A), 19606 (reduced levels of PBP1A expression relative to 17978 [19]) (B), and 19606 LOS^−^ (C) cells. Cells in mid-logarithmic growth were labeled with NADA. (D) Septa were quantified using ImageJ software (*n *= 300) and reported as a percentage of the whole. Data were collected from three experiments, and one representative image and data set were reported. (E) Percentage of LOS^−^ A. baumannii recovered after colistin selection using 10^9^ CFU in logarithmic growth phase.

We examined A. baumannii clinical isolates and found morphological differences between strain ATCC 19606, which produces LOS^−^ populations after colistin selection, and strain ATCC 17978, which cannot (19). Cells in logarithmic growth phase were treated with a fluorescent derivative of d-alanine (NADA) (33), which is incorporated into the peptidoglycan cell wall by PBPs and ld-TPases (34–37). Wild-type 17978 cells were coccobacilli with septal assembly localized at the midcell (Fig. 1A), where septal peptidoglycan synthesis produced two daughter cells during division. In contrast, wild-type 19606 bacteria, which demonstrated an 80-fold reduction in PBP1A expression during mid-logarithmic growth (19), were bacilli containing multiple septal sites (Fig. 1B). LOS^−^ cells derived from 19606 also contained multiple septal sites (Fig. 1C). Unlike 17978, 19606 and LOS^−^ subpopulations contained three or more septa (Fig. 1D), indicating a septation defect. Consistent with previous findings (19), 19606 produced LOS^−^ colonies after colistin selection, whereas 17978 did not (Fig. 1E and Table 1).The average lengths and widths of 17978, 19606, and 19606 LOS^−^ cells were also calculated (Fig. S1A) and showed that wild-type 19606 and 19606-derived LOS^−^ A. baumannii populations contained subsets of elongated cells.

**TABLE 1:** A. baumannii develops colistin resistance through inactivation of lipooligosaccharide (LOS) biosynthesis

A. baumannii strain	Recovery frequency of LOS^**−**^ A. baumannii
Logarithmic growth phase	
*Ab* 5075	2.33E−08
*Ab* 5075 Δ*ldtJ*::Tn	N/A^*a*^
*Ab* 5075 Δ*ldtK*::Tn	N/A
*Ab* ACICU	N/A
*Ab* AYE	8.09E−08
*Ab* SDF	N/A
ATCC 17978	N/A
ATCC 17978 Δ*mrcA*	9.19E−08
ATCC 17978 Δ*mrcA*/pPBP1A	N/A
ATCC 17978 Δ*mrcA*/pPBP1A_S459A_	N/A
ATCC 17978 Δ*mrcA*/pPBP1A_E92Q_	9.19E−08
ATCC 17978 Δ*mrcB*	N/A
ATCC 19606	1.47E−07
ATCC 19606 Δ*ldtJ*	N/A
ATCC 19606 Δ*ldtJ*/pLdtJ	8.99E−06
ATCC 19606 Δ*ldtK*	N/A
ATCC 19606 Δ*ldtK*/pLdtK	2.11E−07
Stationary phase	
*Ab* 5075	1.93E−07
*Ab* ACICU	N/A
*Ab* AYE	9.59E−07
*Ab* SDF	N/A
ATCC 17978	N/A
ATCC 17978 Δ*mrcA*	1.06E−08
ATCC 17978 Δ*mrcA*/pPBP1A	N/A
ATCC 17978 Δ*mrcA*/pPBP1A_S459A_	N/A
ATCC 17978 Δ*mrcA*/pPBP1A_E92Q_	2.01E−08
ATCC 17978 Δ*mrcB*	N/A
ATCC 17978 + 8 g/liter amoxicillin	N/A
ATCC 17978 + 64 g/liter ampicillin	N/A
ATCC 17978 + 4 g/liter carbenicillin	N/A
ATCC 17978 + 32 g/liter mecillinam	N/A
ATCC 17978 + 16 g/liter mezlocillin	N/A
ATCC 17978 + 256 g/liter cefoxitin	N/A
ATCC 17978 + 64 g/liter cefoperazone	N/A
ATCC 17978 + 8 g/liter cefotaxime	N/A
ATCC 17978 + 8 g/liter aztreonam	N/A
ATCC 17978 + 8 g/liter moenomycin	N/A
ATCC 19606	2.13E−07

a N/A indicates that no LOS^−^ isolates were recovered.

10.1128/mBio.02185-20.1 FIG S1 Morphology of A. baumannii isolates in logarithmic growth phase. (A) Cell length (L) and width (W) of wild-type (WT) 17978, 19606, and LOS^−^ isolates derived from 19606 were calculated using ImageJ software (*n *= 300). Each experiment was independently replicated three times; one representative data set was reported in the quantification. Each dot on the graph represents one cell. (B) Percentage of recovered LOS^−^ A. baumannii after colistin selection using 10^9^ CFU in logarithmic growth phase. (C to F) NADA-stained *Ab* 5075 (C), *Ab* AYE (D), *Ab* ACICU (E), and *Ab* SDF (F) were visualized using phase and fluorescent microscopy. A representative image was reported. (G) Septa were quantified using ImageJ software (*n *= 300) and reported as a percentage of the whole. Each experiment was independently replicated three times; one representative data set was reported in the quantification. (H) Cell length (L) and width (W) of wild-type 5075, AYE, ACICU, and SDF were calculated using ImageJ software (*n *= 300). Each experiment was independently replicated three times; one representative data set was reported in the quantification. Each dot on the graph represents one cell. Download FIG S1, TIF file, 1.6 MB. Copyright © 2021 Kang et al. 2021 Kang et al. This content is distributed under the terms of the Creative Commons Attribution 4.0 International license.

To determine if the septation defect was conserved among isolates that support LOS deficiency-mediated colistin resistance, we analyzed several additional A. baumannii clinical isolates. Consistent with strain 19606, *Ab* 5075 (Fig. S1C) and *Ab* AYE (Fig. S1D) also produced multiseptated bacilli and yielded LOS^−^ colistin-resistant isolates (Fig. S1B). In contrast, *Ab* ACICU (Fig. S1E) and *Ab* SDF (Fig. S1F) assembled a single septum at the midcell. As with 17978, we could not recover LOS^−^ isolates from either ACICU or SDF parent strains (Table 1 and Fig. S1B). Septal quantification showed that *Ab* 5075 and *Ab* AYE had subpopulations that produced three or more septa, while *Ab* ACICU and *Ab* SDF did not (Fig. S1G). The average length and width of each clinical isolate were reported (Fig. S1H). Strains showing three or more septa also produced elongated cell subpopulations.

To determine if elongated morphologies were consistent in growth phases, we also analyzed stationary-phase cultures. Only 19606 (Fig. S2A), 5075 (Fig. S2B), and AYE (Fig. S2C) formed filaments and yielded colistin-resistant LOS^−^ isolates in stationary phase (Table 1 and Fig. S2G). 17978 (Fig. S2D), ACICU (Fig. S2E), and SDF (Fig. S2F) maintained a coccobacillus morphology and failed to produce a single LOS^−^ isolate after colistin selection (Table 1 and Fig. S2G). The length and width of each isolate in stationary phase were calculated (Fig. S2H). Strains 19606, *Ab* 5075, and *Ab* SDF were elongated relative to 17978, *Ab* ACICU, and *Ab* AYE. While it was previously shown that only select strains supported isolation of LOS^−^ A. baumannii after colistin selection (19), here we show a correlation between strains with defective septation and selection of colistin-resistant LOS^−^ isolates.

10.1128/mBio.02185-20.2 FIG S2 Microscopy of A. baumannii clinical isolates in stationary phase. (A to F) NADA-stained 19606 (A), *Ab* 5075 (B), *Ab* AYE (C), 17978 (D), *Ab* ACICU (E), and *Ab* SDF (F) were visualized using phase and fluorescent microscopy. A representative image was reported. (G) Percentage of recovered LOS^−^ A. baumannii after colistin selection using 10^9^ CFU in stationary phase. (H) Quantification of length (L) and width (W) of each cell population (*n *= 300) was calculated using ImageJ software. Each experiment was independently replicated three times. One representative data set was reported in the quantification. Each dot on the graph represents one cell. Download FIG S2, TIF file, 1.8 MB. Copyright © 2021 Kang et al. 2021 Kang et al. This content is distributed under the terms of the Creative Commons Attribution 4.0 International license.

### PBP1A mutation induced septal defects and supported isolation of colistin-resistant LOS^**−**^ A. baumannii.

**FIG 2:**
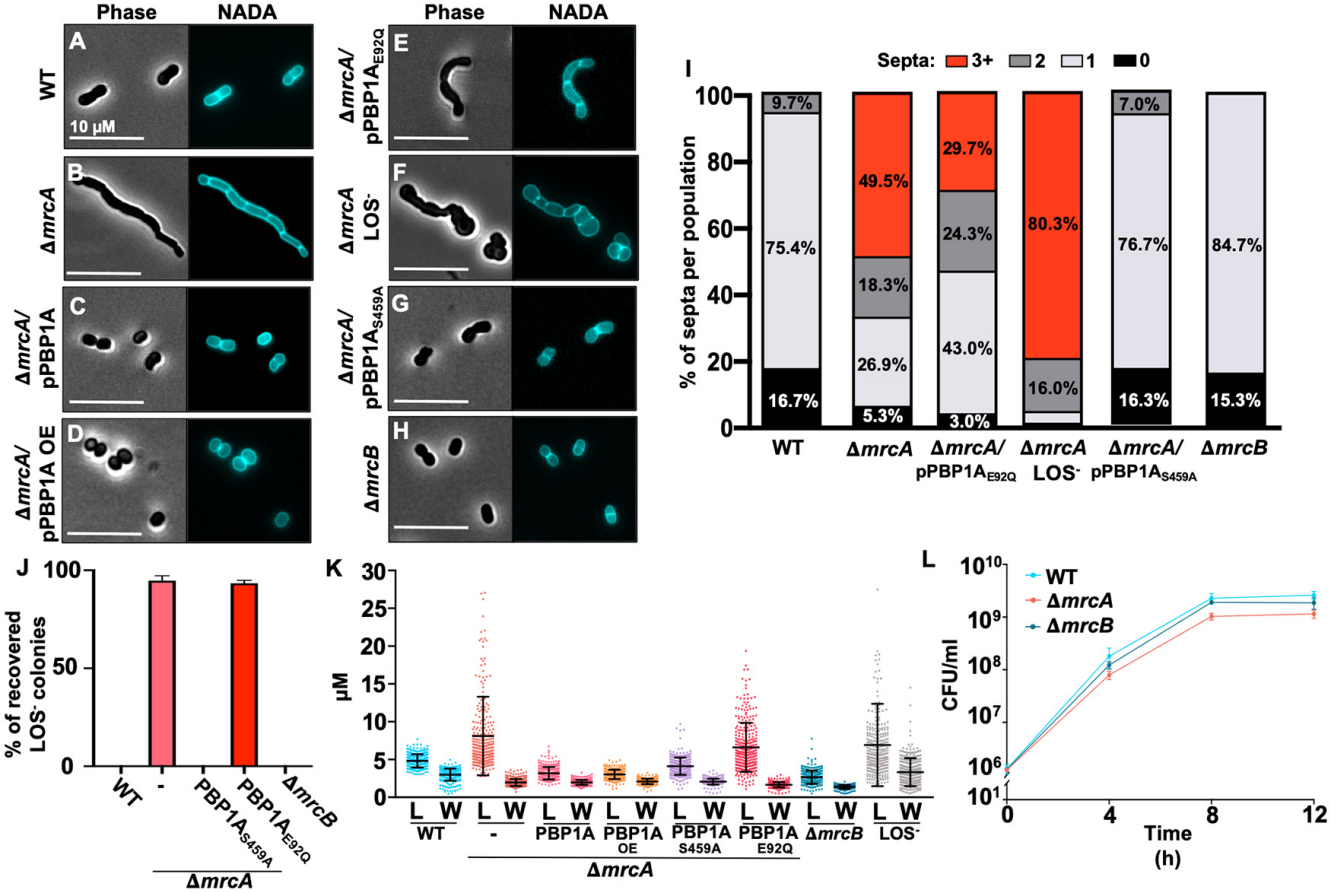
Microscopy of A. baumannii strain 17978 mutants in logarithmic growth phase. (A to H) Phase and fluorescence microscopy of NADA-treated wild-type (WT) (A), Δ*mrcA* (B), Δ*mrcA*/pPBP1A (C), Δ*mrcA*/pPBP1A OE (overexpression) (D), Δ*mrcA*/pPBP1A_E92Q_ (E), Δ*mrcA* LOS^−^ (F), Δ*mrcA*/pPBP1A_S459A_ (G), and Δ*mrcB* (H) cells. (I) Septa were quantified using ImageJ software (*n *= 300) and reported as a percentage of the whole. Each experiment was independently replicated three times, and one representative data set was reported. (J) Percentage of recovered LOS^−^ A. baumannii after colistin selection using 10^9^ CFU in logarithmic growth phase. (K) Quantification of length (L) and width (W) of each cell population (*n *= 300) was calculated using ImageJ software. Each experiment was independently replicated three times, and one representative data set was reported. Each dot on the graph represents one cell. (L) CFU/ml of wild type and aPBP mutants in rich medium at 37°C.

Inactivation of the gene encoding PBP1A, Δ*mrcA*, is a compensatory mutation that enables colistin selection of LOS^−^ 17978 (19). Relative to wild type and two complementation strains with different promoter constructs, the 17978 Δ*mrcA* mutant produced multiseptated cell populations in mid-logarithmic growth phase (Fig. 2A to D), resembling 19606, 5075, and AYE morphotypes. Consistent with previous analysis showing that PBP1A GTase activity inhibited colistin selection of LOS^−^ colonies (19), Δ*mrcA*/pPBP1A_E92Q_ also produced elongated multiseptated cells (Fig. 2E), similarly to Δ*mrcA* LOS^−^ cells (Fig. 2F). In contrast, point mutation of S459A, a residue essential for PBP1A dd-TPase activity (Fig. 2G), and Δ*mrcB* (encoding PBP1B) (Fig. 2H) produced septal patterns indistinguishable from wild-type 17978, ACICU, and SDF. Only PBP1A mutations that induced three or more septal sites (i.e., Δ*mrcA* and Δ*mrcA*/pPBP1A_E92Q_) (Fig. 2I) were sufficient to produce colistin-resistant LOS^−^ isolates (Fig. 2J and Table 1). We also measured the lengths and widths of each mutant (Fig. 2K). Only strains with cell populations containing three or more septal sites were elongated. Relative to wild type, the Δ*mrcA* mutant had a growth defect in logarithmic phase (Fig. 2L) when grown in Luria broth at 37°C. The doubling time was 33.76 ± 3.7 and 36.44 ± 1.9 min in wild-type and Δ*mrcB* strains, respectively, whereas the Δ*mrcA* strain had a slightly slower doubling time of 40.05 ± 2.6 min in logarithmic growth phase.

Unlike wild-type 17978, Δ*mrcA*/pPBP1A_S459A_, and Δ*mrcB* strains (Fig. S3A to C), Δ*mrcA* and Δ*mrcA*/pPBP1A_E92Q_ strains demonstrated a filamentous morphology in stationary phase (Fig. S3D and E) that resembled the Δ*mrcA* LOS^−^ strain (Fig. S3F) and supported recovery of LOS^−^ colistin-resistant isolates (Table 1 and Fig. S3G). The cell lengths, which indicate defective septation, and widths of stationary-phase cultures were measured (Fig. S3H). Consistent with our initial observation that only A. baumannii clinical isolates producing cell populations with three or more septal sites support LOS^−^ selection, PBP1A mutations that induced multiseptate cell morphotypes also supported LOS^−^ isolation. Together, these data indicate that defects in PBP1A-dependent septation correlate with isolation of colistin-resistant LOS^−^ A. baumannii.

10.1128/mBio.02185-20.3 FIG S3 Microscopy of A. baumannii 17978 mutants in stationary phase. (A to F) NADA-stained wild-type (WT) 17978 (A), Δ*mrcA*/pPBP1A_S459A_ (B), Δ*mrcB* (C), Δ*mrcA* (D), Δ*mrcA*/pPBP1A_E92Q_ (E), and Δ*mrcA* LOS^−^ (F) strains were visualized using phase and fluorescent microscopy. One representative image was reported. (G) Percentage of recovered LOS^−^ A. baumannii after colistin selection using 10^9^ CFU in stationary phase. (H) Quantification of length (L) and width (W) of each cell population (*n *= 300) was calculated using ImageJ software. Each experiment was independently replicated three times, and one representative data set was reported. Each dot on the graph represents one cell. Download FIG S3, TIF file, 2.5 MB. Copyright © 2021 Kang et al. 2021 Kang et al. This content is distributed under the terms of the Creative Commons Attribution 4.0 International license.

### PBP1A localizes to the division site in A. baumannii.

**FIG 3:**
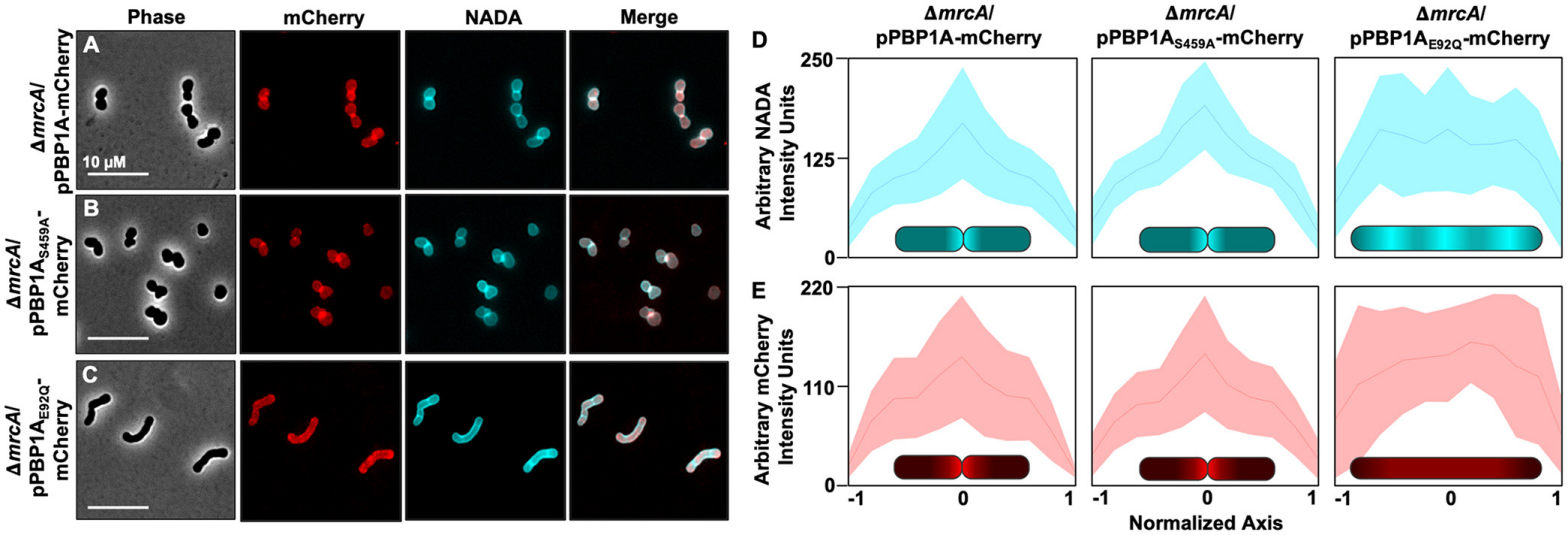
Localization of PBP1A in A. baumannii. (A to C) Phase and fluorescence microscopy of 17978 Δ*mrcA* expressing PBP1A (A), PBP1A_S459A_ (B), or PBP1A_E92Q_ (C) fused to a C-terminal mCherry protein. Cells were labeled with NADA. Merged images are color composites of mCherry and NADA images. (D and E) Localization intensity of NADA (D) and PBP1A-mCherry (E) in cells. Shading indicates standard deviation. Intensity localization graphs generated using ImageJ software with MicrobeJ plugin (*n *= 50). Each experiment was independently replicated three times, and one representative data set was reported. Fluorescence localization intensity within cells is illustrated at the bottom.

Due to the septation defect in Δ*mrcA* and Δ*mrcA*/pPBP1A_E92Q_ strains, we hypothesized that PBP1A contributes to daughter cell formation in A. baumannii. To determine PBP1A localization, we fused mCherry to the C terminus of PBP1A (PBP1A-mCherry) and expressed the construct in Δ*mrcA* cells. Expression levels of each PBP1A-mCherry fusion protein in the Δ*mrcA* mutant were equivalent (Fig. S4A), and PBP1A was required for mCherry signal (Fig. S4B). While mCherry fluorescent signal was observed throughout cells when pPBP1A (Fig. 3A) or pPBP1A_S459A_ (Fig. 3B) fusion proteins were expressed, increased intensity was evident at the midcell, where the septum forms. These findings indicate that PBP1A localizes at the septal site and potentially interacts with the divisome complex in A. baumannii. Phase microscopy showed that pPBP1A-mCherry and pPBP1A_S459A_-mCherry expression fully complemented the Δ*mrcA*-induced division defect to restore the signature A. baumannii coccobacillus morphology. In contrast, pPBP1A_E92Q_-mCherry (Fig. 3C) did not localize at the midcell and cells contained multiple septal sites, showing that GTase-defective PBP1A was not sufficient to complement the Δ*mrcA* mutant.

10.1128/mBio.02185-20.4 FIG S4 Expression of PBP1A-mCherry fusion proteins in A. baumannii 17978. (A) Immunoblot analysis of PBP1A and NADH chain L proteins in whole-cell lysates from wild-type and Δ*mrcA* strains expressing PBP1A-mCherry fusion proteins (PBP1A-mCherry = 121.16 kDa, PBP1A = 94.78 kDa). (B) Phase and fluorescence microscopy of wild-type 17978 carrying an empty vector containing the mCherry coding sequence. Download FIG S4, TIF file, 1.1 MB. Copyright © 2021 Kang et al. 2021 Kang et al. This content is distributed under the terms of the Creative Commons Attribution 4.0 International license.

We also treated cells expressing PBP1A-mCherry proteins with NADA, which is incorporated into the peptidoglycan. pPBP1A-mCherry and pPBP1A_S459A_-mCherry colocalized with septal peptidoglycan at the midcell, but pPBP1A_E92Q_-mCherry did not (Fig. 3A to C). To quantify, intensity localization was graphed along the cell axis (Fig. 3D and E). PBP1A and PBP1A_S459A_ colocalized with septal peptidoglycan, whereas PBP1A_E92Q_ did not. These analyses show not only that the GTase activity of PBP1A is required for proper division in A. baumannii but also that GTase activity is required for PBP1A septal site localization.

### Antimicrobial susceptibility in A. baumannii aPBP mutants.

To determine the impact of aPBP deletions on antimicrobial susceptibility, MICs were calculated after wild type and the aPBP mutants were treated with several antimicrobials (Table 2). Relative to wild type, the Δ*mrcA* mutant showed increased resistance to all β-lactam antibiotics tested except for carbapenems, which target not only dd-TPases but also ld-TPases (38). These data suggest that PBP1A dd-TPase activity is an intrinsic target that contributes to β-lactam susceptibility and that PBP1A has a critical role in cross-linking peptidoglycan. However, dd-TPase activity is not critical for the function of PBP1A that inhibits formation of LOS^−^ cells. Both aPBP mutants showed increased susceptibility to moenomycin, which inhibits GTase activity. No differences in MIC were observed when strains were treated with colistin. Lastly, both aPBP mutants demonstrated increased susceptibility to vancomycin.

**TABLE 2:** A. baumannii MICs

Antimicrobial	MIC (mg/liter) for strain:				
	WT	Δ*mrcA*	Δ*mrcB*	Δ*ldtJ*	Δ*ldtK*
β-Lactams					
Penicillin derivatives					
Amoxicillin	16.0	>512.0	<8.0	128.0	128.0
Ampicillin	128.0	>1,024.0	128.0	16.0	16.0
Carbenicillin	8.0	>64.0	8.0	4.0	4.0
Mecillinam	64.0	>512.0	64.0	16.0	16.0
Mezlocillin	32.0	>256.0	32.0	128.0	128.0

Cephalosporin derivatives					
Cefoxitin	>512.0	64.0	16.0	128.0	128.0
Cefoperazone	128.0	>512.0	128.0	>512.0	>512.0
Cefotaxime	8.0	32.0	4.0	16.0	8.0

Carbapenems					
Imipenem	0.3	0.3	0.3	<0.01	<0.01
Meropenem	0.1	0.1	0.1	<0.01	<0.01

Monobactam					
Aztreonam	16.0	2.0	2.0	8.0	8.0

Phosphoglycolipid					
Moenomycin	16.0	2.0	2.0	4.0	4.0

Lipopeptide					
Colistin	1.0	1.0	1.0	1.0	1.0

Glycopeptide					
Vancomycin	>512.0	64.0	64.0	64.0	64.0

Metal (mM)					
Copper chloride	5.0	4.5	4.0	3.5	3.0

### Filamentation is not sufficient for isolation of colistin-resistant LOS^**−**^ A. baumannii.

While PBP1A GTase activity is required for proper A. baumannii division, defective division is also correlated with isolation of colistin-resistant LOS^−^ A. baumannii. We next tested if filamentation was sufficient to recover colistin-resistant LOS^−^ A. baumannii. Previous studies showed that treatment with dd-TPase-targeting β-lactams induced filamentation in Gram-negative bacteria (39, 40). Therefore, we treated wild-type 17978 with several β-lactams and moenomycin (Table 2). Wild-type 17978 was grown overnight in 0.5× MICs of each antibiotic, and cultures were treated with NADA to visualize morphological changes (Fig. S5A to J). Amoxicillin, cefoperazone, cefotaxime, aztreonam, moenomycin, and cefoxitin treatment induced filamentation (Fig. S5K), likely because they inhibit dd-TPase or glycosyltransferase activity required for cell division. In contrast, cells treated with ampicillin, carbenicillin, amdinocillin, and mezlocillin formed spheres (Fig. S5K), suggesting the β-lactams target primarily dd-TPases associated with cell elongation. We next performed colistin selection on treated cells to isolate LOS^−^ A. baumannii; however, we were unable to recover LOS^−^ isolates from all treated cultures (Table 1 and Fig. S5L), indicating that filamentation is not sufficient for colistin selection of LOS^−^ A. baumannii.

10.1128/mBio.02185-20.5 FIG S5 Treatment of A. baumannii 17978 with subinhibitory concentrations of PBP-targeting antibiotics. (A to J) NADA-stained 17978 was visualized using phase and fluorescent microscopy after 18 to 22 h of treatment with 0.5× MIC of amoxicillin (Amx) (A), cefoperazone (Cfp) (B), cefotaxime (Ctx) (C), aztreonam (Azt) (D), moenomycin (Mm) (E), cefoxitin (Fox) (F), ampicillin (Amp) (G), carbenicillin (Cb) (H), mecillinam (Amd) (I), or mezlocillin (Mz) (J). A representative image was reported. (K) Quantification of length (L) and width (W) of each cell population (*n *= 300) was calculated using ImageJ software. Each experiment was independently replicated three times, and one representative data set was reported. Each dot on the graph represents one cell. (L) Percentage of recovered LOS^−^ A. baumannii after colistin selection using 10^9^ CFU. Download FIG S5, TIF file, 1.5 MB. Copyright © 2021 Kang et al. 2021 Kang et al. This content is distributed under the terms of the Creative Commons Attribution 4.0 International license.

### Peptidoglycan modifications resulting from PBP1A mutation.

**FIG 4:**
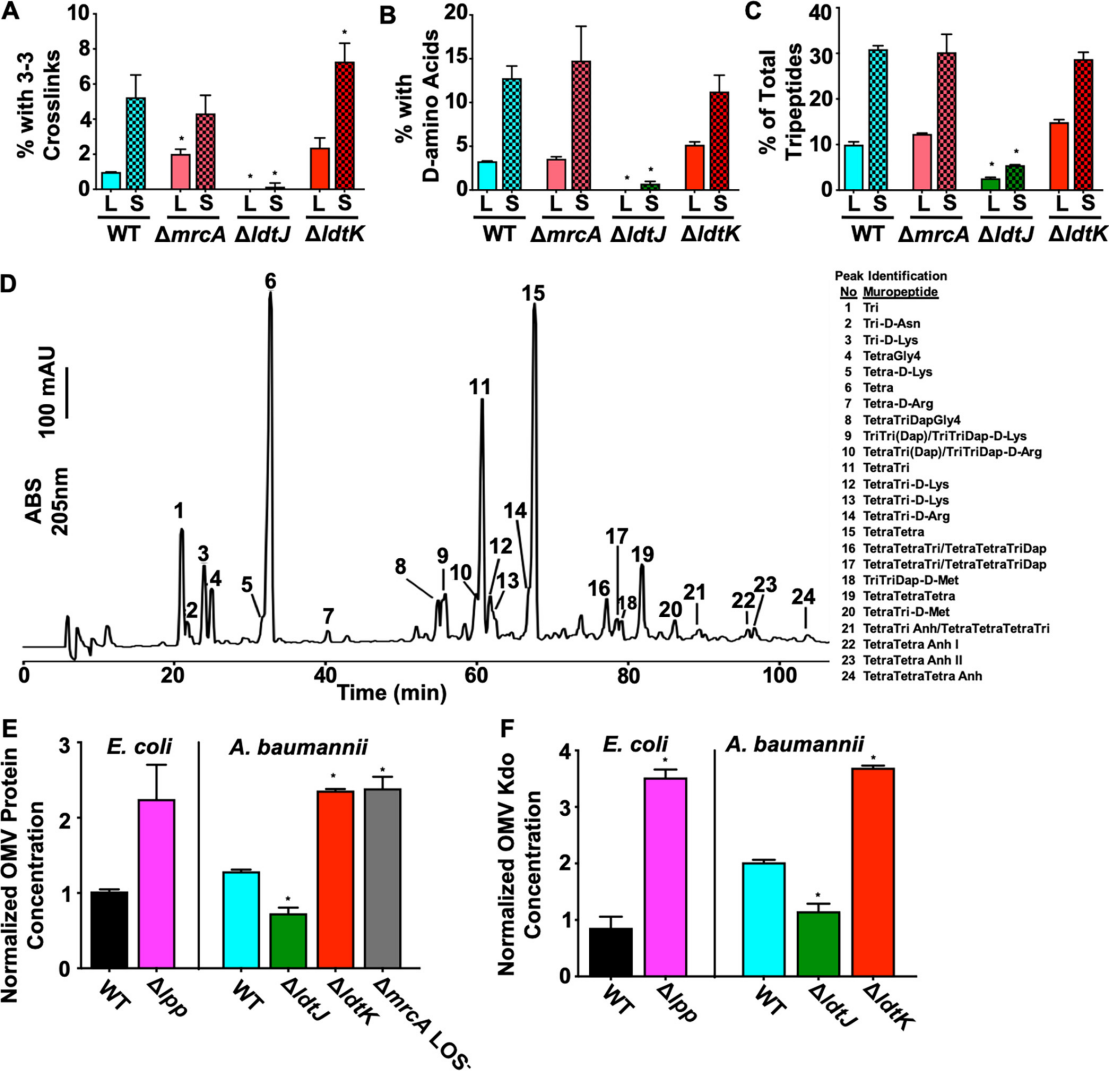
ld-Transpeptidase activity is required for cell envelope modifications in A. baumannii. (A to C) Percentage of total muropeptide content with 3-3 cross-links (A), d-amino acid addition (B), and murotripeptide formation (C). L, logarithmic growth phase; S, stationary phase. Error bars indicate variation of two biological replicates. An asterisk indicates significant differences relative to the corresponding wild-type (WT) strain (*P* < 0.05). (D) Chromatogram of the muropeptide content of stationary-phase 17978 Δ*ldtK.* The muropeptides are labeled. (E and F) Relative quantification of E. coli and A. baumannii total protein (E) and Kdo (F) concentrations of outer membrane vesicles (OMVs) in Δ*ldtJ* and Δ*ldtK* strains relative to wild-type (WT) A. baumannii and E. coli. WT E. coli was normalized to 1. Each experiment was independently replicated three times, and one representative data set was reported. Error bars indicate standard deviations. An asterisk indicates significant differences relative to the corresponding WT strain (*P* < 0.05).

Since we found that the Δ*mrcA* mutant showed increased resistance against several dd-TPase-targeting β-lactam antibiotics, we next sought to determine if the mutation altered the muropeptide composition in logarithmic and stationary phase. Muropeptide compositions of both wild-type and Δ*mrcA* strains were analyzed (Table S1A) and showed increased peptidoglycan modifications in stationary phase relative to logarithmic growth phase. Specifically, 3-3 cross-linking and incorporation of d-amino acids increased (Fig. 4A and B). Both modifications are characteristic of increased ld-TPase activity, which is growth phase dependent in E. coli (41). We also found that relative to wild type, the Δ*mrcA* strain generated 2-fold more 3-3 cross-links in logarithmic growth phase (Fig. 4A and Table S1A).

10.1128/mBio.02185-20.7 TABLE S1 Muropeptide composition of wild-type and mutant A. baumannii strains ATCC 17978 (A) and ATCC 19606 (B). Download Table S1, DOCX file, 0.03 MB. Copyright © 2021 Kang et al. 2021 Kang et al. This content is distributed under the terms of the Creative Commons Attribution 4.0 International license.

### ld-TPases are essential for selection of colistin-resistant LOS^**−**^ A. baumannii.

**FIG 5:**
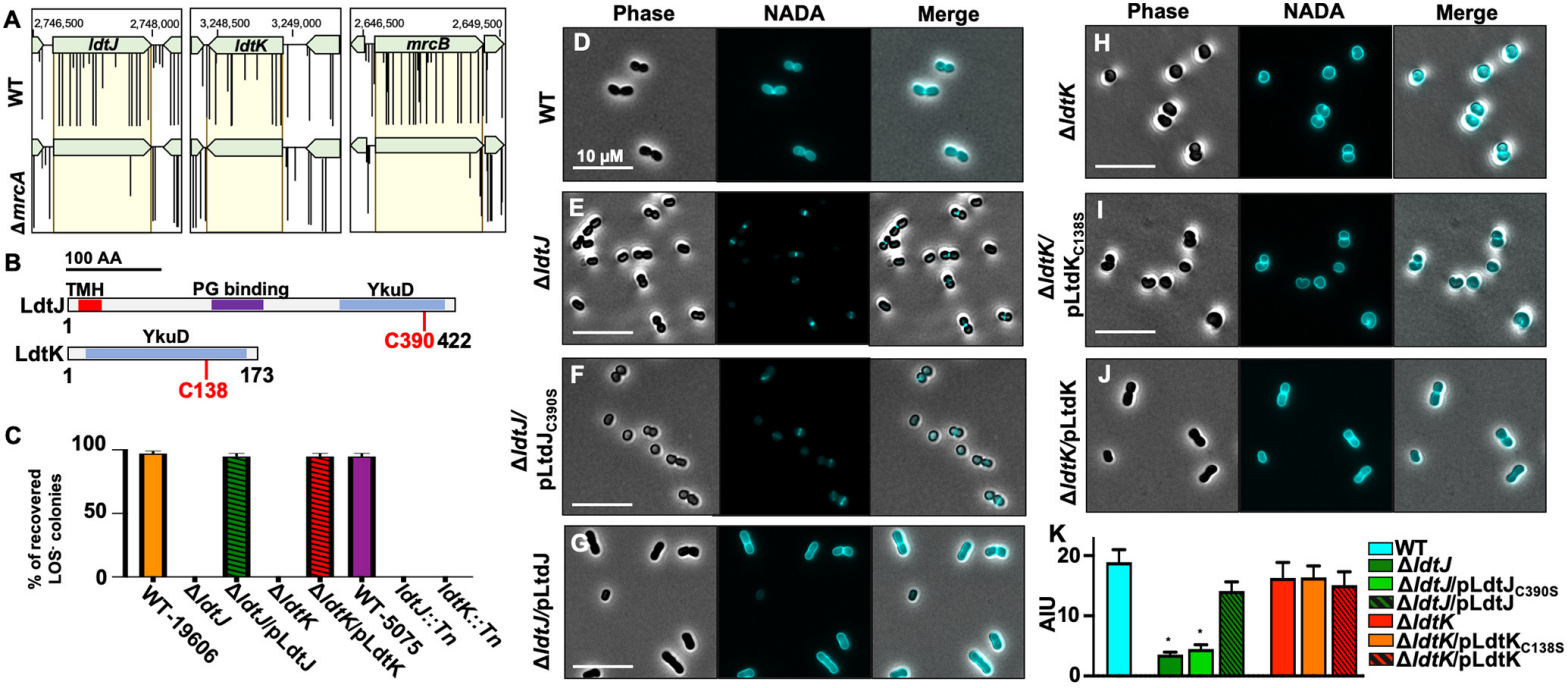
ld-Transpeptidases are required for isolation of LOS^−^ A. baumannii. (A) Transposon sequencing analysis of *ldtJ*, *ldtK*, and *mrcB* in wild-type (WT) and Δ*mrcA* 17978 A. baumannii. Yellow-highlighted areas indicate genes of interest. Black lines indicate transposon insertion sites and abundance. (B) Schematic of domain organization of LdtJ and LdtK. (C) Percentage of recovered LOS^−^ colonies after colistin selection of wild-type and mutant 19606 and *Ab* 5075 strains in logarithmic phase. (D to J) Phase and fluorescence microscopy of NADA-treated wild-type and Δ*ldtJ* (D to G) and Δ*ldtK* (H to J) 17978. Each experiment was independently replicated three times, and one representative data set was reported. (K) Quantification of fluorescent signal intensity in arbitrary intensity units (AIU, *n *= 300). Error bars indicate standard deviations. An asterisk indicates significant differences relative to WT (*P* < 0.05).

Next, we performed transposon sequencing in the Δ*mrcA* mutant to determine genes that contribute to fitness relative to wild type. We discovered that two genes encoding putative ld-TPases (LdtJ and LdtK) were essential in the Δ*mrcA* strain but not in wild type (Fig. 5A). As a control, we also show that mutations in *mrcB* were also synthetically lethal in A. baumannii, as previously reported in E. coli (42, 43). Since PBP1A inactivation supports colistin selection of LOS^−^ A. baumannii (19), and mutations of *ldtJ* and *ldtK* are synthetically lethal in the Δ*mrcA* strain, we hypothesized that ld-TPase activity could support viability of LOS^−^ A. baumannii. LdtJ and LdtK both encode YkuD domains, which rely on essential cysteine residues to catalyze ld-TPase reactions (Fig. 5B). To test if LdtJ and LdtK contribute to selection of colistin-resistant LOS^−^ A. baumannii, we engineered *ldtJ* and *ldtK* mutations in strain 19606, which produced LOS^−^ isolates without compensatory *mrcA* mutations (18, 19). Colistin selection of each mutant failed to recover LOS^−^ A. baumannii after multiple attempts (Fig. 5C and Table 1), showing each putative ld-TPase gene is required for LOS^−^ viability. Complementation fully restored production of LOS^−^ A. baumannii to wild-type levels. Furthermore, we also performed colistin selection experiments using *ldtJ* and *ldtK* mutants in strain *Ab* 5075. These data showed that *Ab* 5075 ld-TPase mutants were also unable to produce LOS^−^ isolates relative to wild type (Fig. 5C and Table 1), which suggests a conserved role for ld-TPases in viability of LOS^−^ A. baumannii.

To determine the ld-TPase activities of LdtJ and LdtK, the mutants were first treated with NADA and visualized. Δ*ldtJ* (Fig. 5E) and Δ*ldtJ*/pLdtJ_C390_ (Fig. 5F) strains showed a severe defect in NADA incorporation relative to wild type (Fig. 5D) and the mutant complemented with a wild-type allele (Fig. 5G), suggesting LdtJ is an ld-TPase that modifies peptidoglycan with d-amino acids. Δ*ldtK* (Fig. 5H) and Δ*ldtK*/pLdtK_C138S_ (Fig. 5I) strains showed a rounded cell morphology relative to wild type (Fig. 5D) and the complemented strain (Fig. 5J), suggesting a role for LdtK in elongation. Fluorescence intensity (Fig. 5K) and cell shape (Fig. S6A) from NADA-treated cultures were quantified. The ld-TPase mutants were also defective in growth in Luria broth at 37°C relative to the wild-type and complemented strains (Fig. S6B).

10.1128/mBio.02185-20.6 FIG S6 Characterization of ld-transpeptidase mutants. (A) Length (L) and width (W) quantifications of wild-type (WT) and mutant cell populations (*n *= 300). Values were calculated using ImageJ software. Each experiment was independently replicated three times, and one representative data set was reported. Each dot on the graph represents one cell. (B) Growth curve of wild type and mutants in rich medium at 37°C. (C) ProQ4000 Emerald Gold staining of proteinase K-treated whole-cell lysates. (D) ^32^P-radiolabeled lipid A isolated from wild-type and mutant 17978 strains. Download FIG S6, TIF file, 0.9 MB. Copyright © 2021 Kang et al. 2021 Kang et al. This content is distributed under the terms of the Creative Commons Attribution 4.0 International license.

### LdtJ forms 3-3 cross-links and incorporates d-amino acids into peptidoglycan.

To define ld-TPase-dependent peptidoglycan modifications, we isolated peptidoglycan from strains 17978 (Table S1A) and 19606 (Table S1B). Muropeptides were generated by treatment with muramidase, separated by high-performance liquid chromatography, and if necessary, analyzed by tandem mass spectrometry (MS/MS) (Table S2) (19, 44). Peptidoglycan composition from the Δ*ldtJ* mutant showed it was unable to generate 3-3 cross-links (Fig. 4A) or incorporate fluorescent d-amino acids along the lateral cell wall (Fig. 4B and Fig. 5E). The Δ*ldtJ* strain also had reduced pools of murotripeptides (Fig. 4C) with a concomitant increase in murotetrapeptide abundance (Table S1A). Peptidoglycan isolated from stationary-phase Δ*ldtJ* cells showed similar structures, where 3-3 cross-linking, fluorescent d-amino acid incorporation along the lateral cell wall, and murotripeptide pools were significantly reduced relative to wild type (Fig. 4A to C). Similar trends were also found in 19606 (Table S1B). These studies indicate that LdtJ is an ld-TPase required for 3-3 cross-link formation in A. baumannii, while its presence also promotes ld-carboxypeptidase activity. While a previous report showed that d-Lys is incorporated into peptidoglycan of some strains of A. baumannii during stationary phase to protect the cell from effector proteins (44), here we showed via MS/MS that d-Asn, d-Arg, and d-Met are also incorporated into A. baumannii peptidoglycan via LdtJ activity in stationary phase (Fig. 4D and Table S1A and B).

10.1128/mBio.02185-20.8 TABLE S2 Reduced muropeptides from Acinetobacter baumannii 17978 Δ*ldtK*. Download Table S2, DOCX file, 0.01 MB. Copyright © 2021 Kang et al. 2021 Kang et al. This content is distributed under the terms of the Creative Commons Attribution 4.0 International license.

### LdtK regulates outer membrane vesiculation.

In contrast to the Δ*ldtJ* mutant, the muropeptide composition of the Δ*ldtK* mutant showed slight increases of d-amino acid modification, 3-3 cross-linking, and murotripeptide pools relative to wild type in logarithmic growth phase (Fig. 4A to D and Table S1A and B), indicating that LdtJ activity may increase in the absence of LdtK. We did not observe direct changes to LOS (Fig. S6C) or lipid A (Fig. S6D) structures in either the Δ*ldtJ* or Δ*ldtK* mutant. However, we found that the Δ*ldtK* strain formed significantly more outer membrane vesicles (OMVs) than the wild type when total outer membrane vesicle protein content (Fig. 4E) or 3-deoxy-d*-manno*-oct-2-ulosonic acid (Kdo) concentrations (Fig. 4F) were quantified from outer membrane vesicles. In E. coli, LdtABC catalyzes transpeptidation between the outer membrane-anchored Braun’s lipoprotein (Lpp) to peptidoglycan stem peptides, which stabilize the outer membrane lipid bilayer (30). We found that deletion of Lpp in E. coli resulted in hypervesiculation of the outer membrane, similar to Δ*ldtK* A. baumannii (Fig. 4E and F). These studies suggest that LdtK functions to stabilize the outer membrane, possibly by linking it to other structures within the cell envelope. Interestingly, LOS^−^ A. baumannii also produced significantly more outer membrane vesicles than wild type (Fig. 4E), suggesting the LOS-deficient outer membrane is unstable. Therefore, mechanisms that stabilize the outer membrane and cell envelope likely contribute to LOS^−^ cell viability.

## DISCUSSION

The molecular factors that support A. baumannii survival without LOS are not well understood. LOS^−^ A. baumannii assembles multiple septal sites to produce cell chains or filaments. A previous study found that PBP1A GTase activity inhibited isolation of colistin-resistant LOS^−^ A. baumannii (19). Here, we showed that PBP1A GTase activity, which is presumably also required for its TPase activity, is required for proper cell division in A. baumannii. GTase inactivation resulted in septal site accumulation that correlated with selection of LOS^−^ A. baumannii. E. coli cells are also known to form multiseptated chains upon LptC depletion or treatment with the LpxC inhibitor LPC-058, which compromise LPS transport and biosynthesis, respectively (29). Together, these studies support a model where septal defects support Gram-negative bacterial survival when LPS/LOS assembly and/or localization is compromised. Since we know outer membrane biogenesis limits the rate of LOS^−^ A. baumannii growth (45), slowed septation via PBP1A mutation could reduce the growth rate enough to support LOS^−^ outer membrane biogenesis. Even more intriguing is the idea that increased formation of septal sites supports LOS^−^ A. baumannii growth through outer membrane stabilization. In E. coli, transenvelope complexes with the Tol system and CpoB promote outer membrane constriction and septum peptidoglycan cleavage (23, 46, 47). While LPS/LOS is the major stabilizing factor in the outer membrane (13, 48), we do not understand how the lipid bilayer remains intact when LPS/LOS is compromised. However, increasing outer membrane attachment sites via septal site accumulation could increase LOS^−^ outer membrane stability by directly linking it to other components within the cell envelope.

Cell division in E. coli is facilitated by the divisome complex, which includes more than 20 essential and accessory proteins that facilitate and regulate septal peptidoglycan synthesis, constrict the cell envelope, and separate the daughter cells. Divisome assembly is an ordered process where the Z-ring initially recruits class A PBPs via the FtsZ membrane anchors, FtsA-FtsN and ZipA, for a preseptal phase of peptidoglycan synthesis (49). Only after some delay, the next cell division proteins are recruited, including FtsQLB, FtsW-PBP3, and FtsN, which are required for constriction and daughter cell separation (50, 51). PBP1B associates with ZipA and FtsN (the latter interacts with FtsA) during the preseptal phase and later with FtsW/PBP3 and PBP3 (23, 24), and both its GTase and dd-TPase activities are stimulated by LpoB (46, 52, 53) and FtsN (54, 55).

Cell division in A. baumannii has not been well studied; however, we showed that the GTase activity of PBP1A promotes daughter cell separation. In E. coli, PBP1A localizes at the midcell in the absence of PBP1B, where it appears to compensate during division (26). Therefore, it is not unreasonable to suggest a primary role for PBP1A in septation in A. baumannii. Based on the formation of viable but chained A. baumannii in the *pbp1A* mutant, PBP1A GTase activity likely plays a role additional to FtsW-PBP3 in septal peptidoglycan synthesis. Since the PBP1A_E92Q_ mutant forms a septum and constricts, but daughter cell separation is delayed, the GTase activity is likely required for the function of hydrolases, as shown in E. coli (56). Hence, PBP1A-mediated *de novo* peptidoglycan synthesis could be required for producing peptidoglycan that is remodeled by the hydrolases, which could couple peptidoglycan synthesis with daughter cell separation. Moreover, it appears as if GTase activity is required for PBP1A localization at the midcell in A. baumannii. PBP1A interaction with FtsW/PBP3 could be disrupted in the mutant, inhibiting septal localization. In E. coli, PBP1B GTase activity is regulated by many proteins (LpoA, FtsN, FtsW/PBP3, FtsQLB, and PgpB [57]), and so disruption of activity may result in mislocalization because it cannot associate with a specific regulatory factor. Further investigation is needed to understand if PBP1A directly contributes to septation via interactions with divisome components.

Since PBP1A inactivation supports colistin selection of LOS^−^ A. baumannii (19), and *ldtJ* and *ldtK* are synthetically lethal in the Δ*mrcA* mutant, each ld-TPase activity likely fortifies the cell envelope to support LOS^−^ A. baumannii growth. Furthermore, the Δ*mrcA* strain demonstrated a significant increase in 3-3 cross-linking relative to wild type (Fig. 4A). We showed that LdtJ is required for incorporation of d-amino acids, 3-3 cross-linking, and increased carboxypeptidase activity, while *ldtK* mutation decreased outer membrane stability. These distinct phenotypes indicate that separate ld-TPase activities coordinate to stabilize the cell envelope. LdtJ and LdtK have homology to E. coli LdtD and LdtB, respectively. LdtD works in complex with PBP1B and a d-alanyl-d-alanine carboxypeptidase (PBP6a) to increase the ratio of 3-3 to 4-3 cross-links, a mechanism that fortifies the cell envelope to enable E. coli survival when LPS transport is disrupted (29). LdtB catalyzes covalent linkage of outer membrane-anchored Braun’s lipoprotein (Lpp) to peptidoglycan (32), while other Ldts covalently link outer membrane proteins to peptidoglycan (58, 59), which both presumably stabilize the cell envelope. Furthermore, our data indicate that LdtJ and LdtK could contribute comparable enzymatic activities in A. baumannii. Together, inactivation of PBP1A could support LOS^−^ A. baumannii growth by inducing a septation defect, peptidoglycan remodeling, and cross-linking of outer membrane lipoprotein to peptidoglycan to collectively stabilize the cell envelope when the major outer membrane-stabilizing factor, LPS/LOS, is compromised.

Intrinsic antimicrobial resistance is not well understood in A. baumannii, but our analysis indicates that PBP1A is an important component for β-lactam susceptibility. Our antimicrobial susceptibility studies suggest PBP1A dd-TPase activity contributes to 4-3 cross-links in both the Rod-complex and divisome, where the Δ*mrcA* mutant showed increased susceptibility to Rod-complex- and divisome-targeting β-lactams. This is further supported by our PBP1A localization studies, where PBP1A is enriched at the midcell, where the divisome assembles, but also localizes along the lateral cell wall (Fig. 3), where the Rod-complex regulates peptidoglycan insertion to elongate rod-shaped bacteria. However, further studies are necessary to determine PBP1A contributions to both division and elongation in A. baumannii.

ld-TPases are targeted by carbapenems (38) and copper chloride (60), and we observed altered susceptibility to both in the *ldtJ* and *ldtK* mutants (Table 2). While we performed these studies in the single ld-TPase mutants, we were not able to engineer an *ldtJ ldtK* double mutant after several attempts, indicating that one of the genes may be required for A. baumannii survival. A more detailed analysis is needed to characterize the LdtJ and LdtK proteins to understand their contribution to resistance against clinically important antimicrobial compounds, which will inform more effective treatment strategies to combat A. baumannii infections.

## MATERIALS AND METHODS

### Bacterial strains and growth.

All strains and plasmids used in this study are listed in Table S3 in the supplemental material. All A. baumannii strains were grown from freezer stocks initially on Luria-Bertani (LB) agar at 37°C. For selection, 7.5 μg/ml of kanamycin or 10 μg/ml of colistin was used when appropriate. Strains that harbored the pABBRKn plasmid for complementation or overexpression were supplemented with 30 μg/ml of kanamycin.

10.1128/mBio.02185-20.9 TABLE S3 Strains and plasmids used in this study. Download Table S3, DOCX file, 0.03 MB. Copyright © 2021 Kang et al. 2021 Kang et al. This content is distributed under the terms of the Creative Commons Attribution 4.0 International license.

### Construction of PBP1A-mCherry fusions and overexpression vector.

Primers used in this study are listed in Table S4. The mCherry2B gene was amplified from pMV261 (61), purified, and cloned into the KpnI and SacI sites of pABBRKn (19) to generate pABBRKn::mCherry. The *mrcA* coding sequence (encoding PBP1A) from A. baumannii strain ATCC 17978 was amplified from plasmids pPBP1A, pPBP1A_E92Q_, and pPBP1A_S459A_ (19); purified; and cloned into the XhoI and SacI sites in pABBRKn::mCherry, to create pPBP1A-mCherry, pPBP1A_E92Q_-mCherry, and pPBP1A_S459A_-mCherry, respectively. Plasmids were transformed into the Δ*mrcA* mutant for localization studies.

10.1128/mBio.02185-20.10 TABLE S4 Primers used in this study. Download Table S4, DOCX file, 0.01 MB. Copyright © 2021 Kang et al. 2021 Kang et al. This content is distributed under the terms of the Creative Commons Attribution 4.0 International license.

To construct the inducible pPBP1A vector, the *mrcA* coding sequence (encoding PBP1A) was amplified from A. baumannii strain ATCC 17978 cDNA, digested with KpnI and SalI restriction enzymes, and cloned into pMMB67EHKn. The plasmid was transformed into the Δ*mrcA* strain and induced with 2 mM isopropyl-β-d-thiogalactopyranoside (IPTG) for overexpression studies.

### Construction of ld-transpeptidase genetic mutants.

All A. baumannii mutations were isolated as previously described (62). Briefly, REC_Ab_ (pAT03) was expressed in A. baumannii ATCC 17978 or 19606. A linear PCR product containing the FLP recombination target (FRT)-flanked kanamycin resistance cassette with flanking 125-bp regions of homology to either *ldtJ* or *ldtK* was transformed. Transformants were recovered in Luria broth, collected via centrifugation, and plated on LB supplemented with kanamycin. PCR and Sanger sequencing verified all genetic mutations.

Removal of the pMMB67EH::REC_Ab_ Tet^r^ plasmid following isolation of mutants was performed as previously described (63). pMMB67EH carrying the FLP recombinase was transformed into cured mutants. Cells were recovered in Luria broth and plated on LB agar supplemented with IPTG to induce expression of the FLP recombinase. PCR was used to confirm excision of the kanamycin cassette.

For complementation, *ldtJ* or *ldtK* coding sequences were cloned into the XhoI and KpnI sites in pMMB67EHKn (19). Plasmids were transformed into the respective mutant to complement. For site-directed mutagenesis, complementation plasmids were amplified with primers to change the active-site cysteine residue to serine. Constructs were confirmed by Sanger sequencing and transformed into the respective mutant. A. baumannii mutants expressing complementation plasmids were grown in 2 mM IPTG to induce expression.

### Isolation of LOS^**−**^ A. baumannii and determination of mutation frequency.

Isolation of LOS^−^ A. baumannii colonies was done as previously described (19) with slight alterations. Briefly, cultures were grown to mid-logarithmic growth phase or stationary phase with or without antibiotics. One milliliter of optical density at 600 nm (OD_600_) of 1.0 (∼10^9^ CFU) was collected via centrifugation at 1,500 × *g*. Cells were washed with 1 ml of Luria broth and plated on LB agar supplemented with 10 μg/ml of colistin. Isolated colonies were picked and replica plated on LB agar supplemented with vancomycin (10 μg/ml) and LB agar supplemented with colistin (10 μg/ml). Colonies sensitive to vancomycin but resistant to colistin were deemed LOS deficient.

Determination of the mutation frequency was done as previously described (19). The mutation frequency was calculated for three biological replicates, and one representative set was reported.

### Western blotting.

Western blot analysis was carried out via gel transfer to polyvinylidene difluoride (PVDF) (Thermo Fisher Scientific). All blots were blocked in 5% milk for 2 h. The primary antibodies anti-PBP1A (α-PBP1A) and α-NADH chain L were used at 1:1,000 and 1:500 (19), respectively, followed by α-rabbit-horseradish peroxidase (HRP) secondary antibody at 1:10,000 (Thermo Fisher Scientific). SuperSignal West Pico Plus (Thermo Fisher Scientific) was used to measure relative protein concentrations.

### Peptidoglycan analysis.

Biological replicates were grown to either stationary or mid-logarithmic growth phase in 400 ml LB. Cells were collected at 4°C, suspended in 6 ml chilled 1× phosphate-buffered saline (PBS), and lysed with dropwise addition to 6 ml boiling 8% SDS. Peptidoglycan was prepared from cell lysate as previously described (64). Briefly, muropeptides were released from peptidoglycan by the muramidase Cellosyl (Hoechst, Frankfurt am Main, Germany), reduced by sodium borohydride, and separated on a 250- by 4.6-mm 3-μm Prontosil 120-3-C_18_ AQ reversed-phase column (Bischoff, Leonberg, Germany). The eluted muropeptides were detected by absorbance at 205 nm. Eluted peaks were assigned based on published chromatograms (19, 44); new peaks were subjected to MS/MS analysis. Peak means and variation from two independent biological repeats were reported for all samples.

### Fluorescent NADA staining.

Overnight cultures were back-diluted to an OD_600_ of 0.05 and grown at 37°C in LB medium until they reached stationary or mid-logarithmic growth phase. Cells were washed once with Luria broth and resuspended in 1 ml Luria broth. Three microliters of 10 mM NBD-(linezolid-7-nitrobenz-2-oxa-1,3-diazol-4-yl)-amino-d-alanine (NADA) (Thermo Fisher) was added to the resuspension. Cells were incubated with NADA at 37°C for 30 min. Following incubation, cells were washed once and fixed with 1× phosphate-buffered saline containing a (1:10) solution of 16% paraformaldehyde.

### Microscopy.

Fixed cells were immobilized on agarose pads and imaged using an inverted Nikon Eclipse Ti-2 widefield epifluorescence microscope equipped with a Photometrics Prime 95B camera and a Plan Apo 100× 1.45-numerical-aperture lens objective. Green fluorescence and red fluorescence images were taken using a filter cube with 470/40-nm or 560/40-nm excitation filters and 632/60 or 535/50 emission filters, respectively. Images were captured using NIS Elements software.

### Image analysis.

All microscopy images were processed and pseudocolored with ImageJ Fiji (65). A cyan lookup table was applied to NADA images, and a red lookup table was applied to mCherry images. The MicrobeJ plugin was used for quantifications (66). Cell lengths, widths, and fluorescence intensities as a function of length were quantified in MicrobeJ. Cell length, width, and fluorescence data were plotted in Prism 8 (GraphPad 8.4.1). NADA stain was pseudocolored using the MicrobeJ cyan lookup table. Phase and fluorescent channels were merged in MicrobeJ. Fluorescence localization graphs of dividing cells were generated using MicrobeJ XStatProfile. MicrobeJ feature detection was used to calculate the number of septal sites per cell stained with NADA as described above. Septal site percentages were represented with dot plots generated in Prism. Fifty cells were analyzed for fluorescent localization, and 300 cells were analyzed for all other experiments. Each experiment was independently replicated three times, one representative data set was reported in the quantification ,and one representative image was included in the figure.

### Optical density growth curves.

Growth curves were performed as previously described (67). Briefly, overnight cultures were back-diluted to an OD_600_ of 0.01 and set up as triplicate biological replicates in a 96-well plate (BrandTech Brand). A BioTek SynergyNeo^2^ microplate reader was used to record optical density, which was read at OD_600_ every half hour. The microplate reader was set to 37°C with continuous shaking. Growth curves were plotted in Prism 8. Each growth curve experiment was independently replicated three times, and one representative data set was reported.

### CFU growth curve.

Triplicate overnight cultures were diluted back to an OD_600_ of 0.01 and grown for 12 h at 37°C in LB broth. Cells were plated at designated time points on LB agar. LB agar plates were grown overnight at 37°C, and the CFU were enumerated and reported. Growth curves were created in GraphPad Prism 8. Each growth curve experiment was independently replicated twice in triplicate, and one representative data set was reported. Doubling times were calculated using the exponential growth equation *y*(*t*) = *y*0*e^kt^* where *y* is cell density and *k* is growth rate. Standard deviation was calculated from the distribution among the reported data set.

### MIC calculation.

MIC assays were performed as previously described with slight modifications (48, 68). A small number of bacteria from an overnight plate were used to inoculate 5 ml LB at an OD_600_ of 0.05 and grown to mid-logarithmic growth phase. Cells were washed twice with LB medium and diluted to an OD_600_ of 0.01. One hundred fifty microliters of cells was added to each well of a 96-well plate. Antimicrobials and copper chloride (VWR) were diluted in water and serially diluted. Twofold dilutions of each compound were added to each well. Plates were incubated at 37°C overnight with shaking. MICs were determined by OD_600_ measurements where cell density was 0. Each experiment was performed twice in triplicate, and a representative MIC was reported.

### Transposon sequencing.

Transposon sequencing was performed as previously described (69). Briefly, pJNW684 was conjugated into wild-type and Δ*mrcA* A. baumannii strain ATCC 17978. A library of approximately 400,000 mutants was screened for growth in Luria broth. After 6 doublings, genomic DNA (gDNA) from cultures was isolated and sheared, and transposon junctions were amplified and sequenced. Transposon insertions from wild-type and Δ*mrcA* strains were compared to determine factors that influence fitness. The transposon insertion maps for *ldtJ* and *ldtK* genes in wild-type and Δ*mrcA* strains were reported.

### Outer membrane vesicle isolation.

Overnight cultures were back-diluted to an OD_600_ of 0.01 and grown to stationary phase in 100 ml Luria broth as biological duplicates. Cultures were pelleted, and the supernatant was filtered through an 0.45-μm filter (Fisherbrand). Equivalent volumes of filtered supernatant were subjected to ultracentrifugation (Sorvall WX 80+ ultracentrifuge with AH-629 swinging bucket rotor) at 4°C for 1 h and 151,243 × *g*. The outer membrane vesicle pellet was resuspended in 500 μl of cold buffer (50 mM Tris, 5 mM NaCl, 1 mM MgSO_4_; pH 7.5). Outer membrane vesicles from each strain were isolated three times in biological duplicates.

### Quantification of outer membrane vesicles.

For Bradford assays, a standard curve was prepared from dilution of bovine serum albumin (0 to 20 mg/ml) in Pierce Coomassie Plus assay reagent (ThermoFisher) to a final volume of 1 ml. In parallel, outer membrane vesicles (15, 20, and 30 μl) were diluted in reagent to a final volume of 1 ml. Absorbance (OD_595_) was measured in a 96-well plate (BrandTech) using a microplate spectrophotometer (Fisherbrand AccuSkan). Optical densities of samples were compared to the standard curve plotted in Excel (Microsoft), and quantifications were graphed in Prism 8. Experiments were reproduced three times from each outer membrane vesicle isolation, and one representative data set was reported.

Kdo assays were performed as previously described (70). Briefly, 0 to 100 μg/ml Kdo (Sigma) standards were diluted in parallel with isolated outer membrane vesicles (2, 5, 8, and 10 μl) in 0.5 M H_2_SO_4_ (Sigma). Outer membrane vesicle (OMV) isolates were boiled for 10 min. An 0.1 M concentration of periodic acid (Sigma), 0.2 M sodium arsenite (Sigma) in 0.5 M HCl (Sigma), and 0.6% thiobarbituric acid (Sigma) were incubated with Kdo standards and OMV isolates. All samples were boiled, and *n*-butanol (Sigma) was used to extract the purified Kdo prior to optical density measurements taken at OD_552_ and OD_509_ (Fisherbrand AccuSkan microplate spectrophotometer) in cuvettes (Fisherbrand). Readings at OD_552_ were subtracted from OD_509_ and used to generate a linear Kdo standard curve in Excel (Microsoft). Optical densities of samples were compared to the standard curve to quantify. Values were graphed in Prism 8. Each experiment was reproduced three times from each outer membrane vesicle isolation, and one representative data set was reported.

### Statistical analysis.

Tests for significance in differences of muropeptide composition and outer membrane vesicle production were conducted using the Student *t* test (two-tailed distribution with two-sample, equal variance calculations). Statistically significant differences between relevant strains possessed *P < *0.05.
