# Supplementary figures and images for: Septal Class A Penicillin-Binding Protein Activity and ld-Transpeptidases Mediate Selection of Colistin-Resistant Lipooligosaccharide-Deficient Acinetobacter baumannii
Source: mBio. 2021 Jan 5;12(1):e02185-20. doi: 10.1128/mBio.02185-20 (PMC8545086; doi:10.1128/mBio.02185-20)

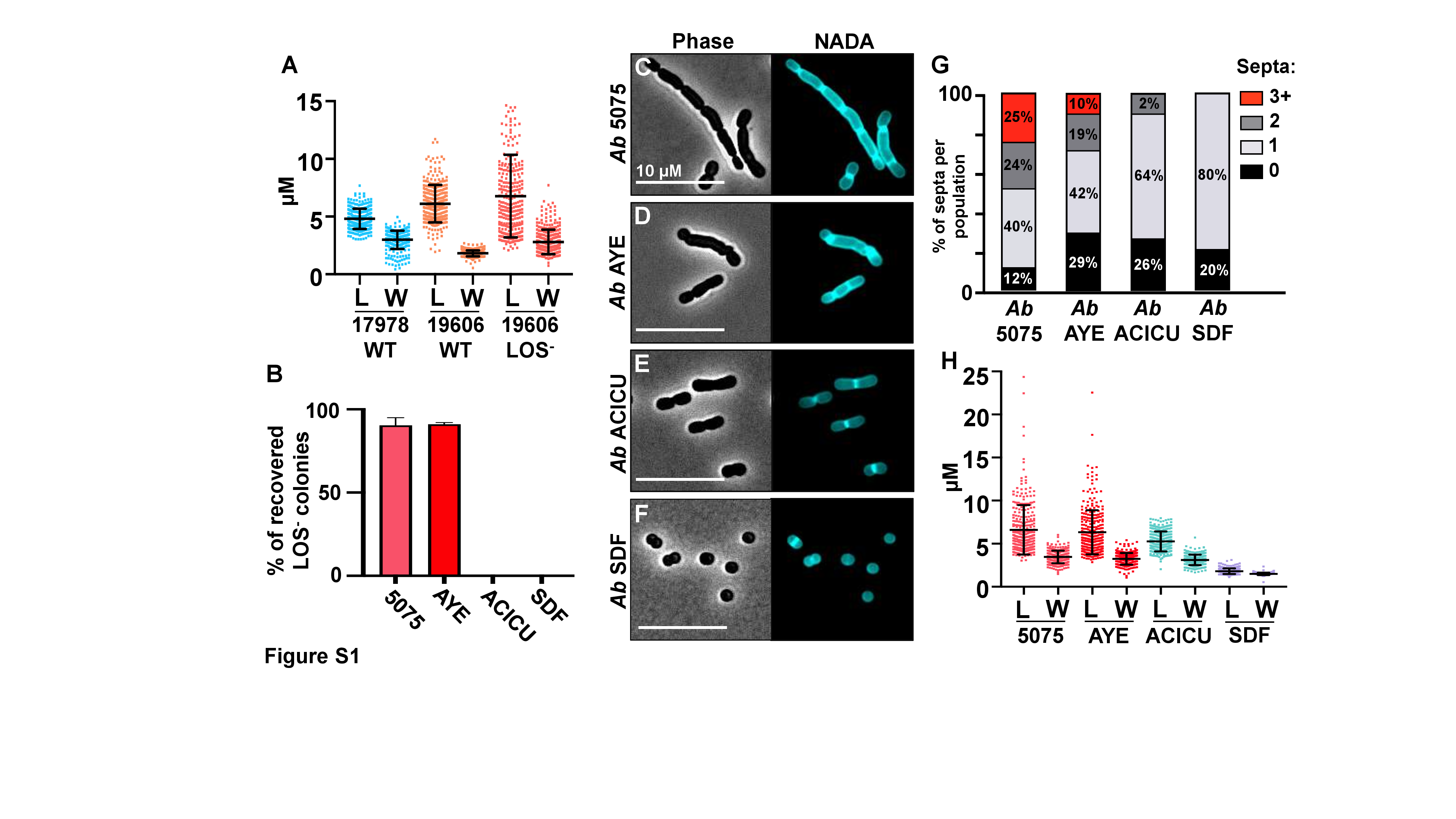

Supplement: FIG S1 [file mbio.02185-20-sf001.tif]

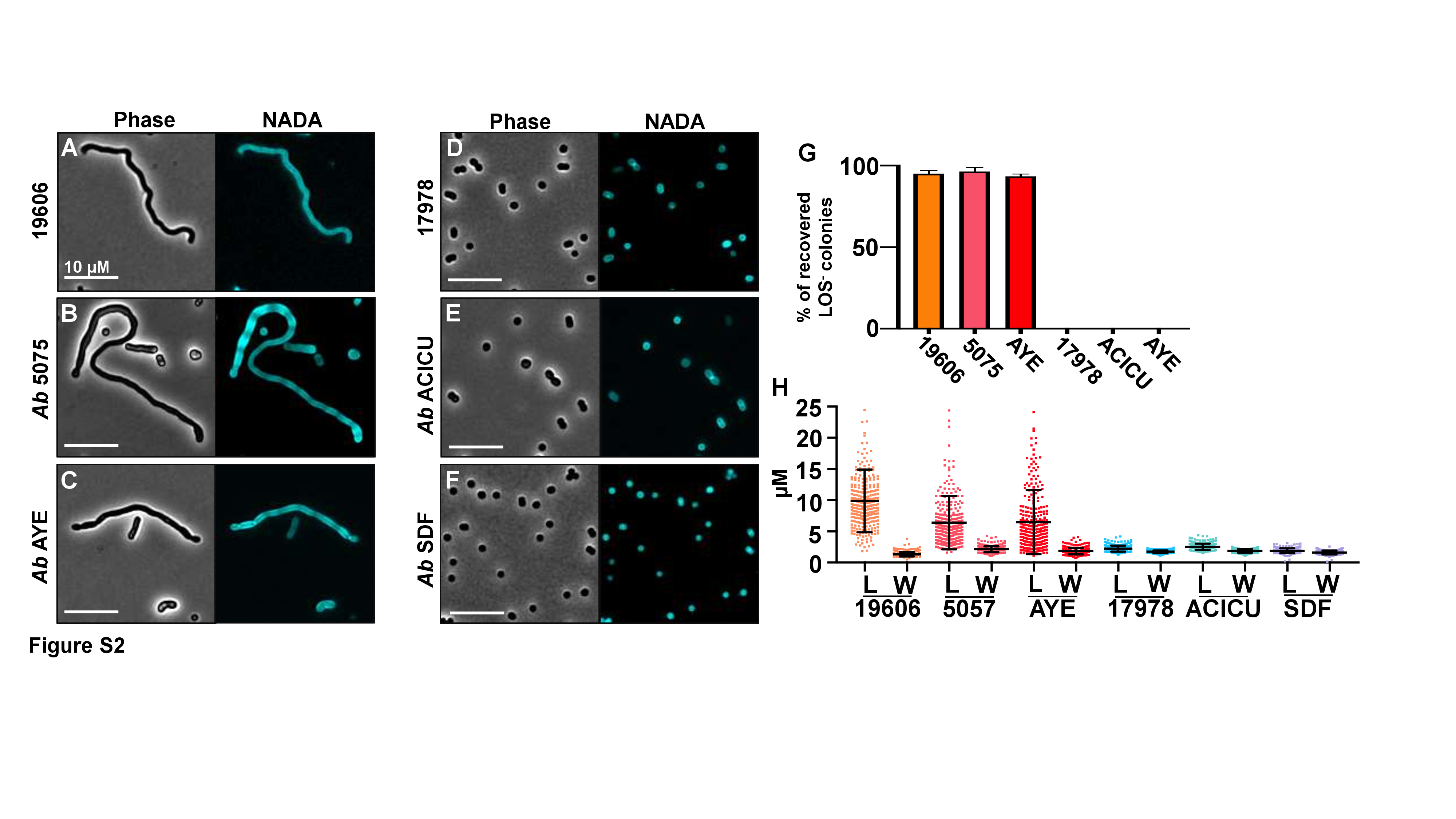

Supplement: FIG S2 [file mbio.02185-20-sf002.tif]

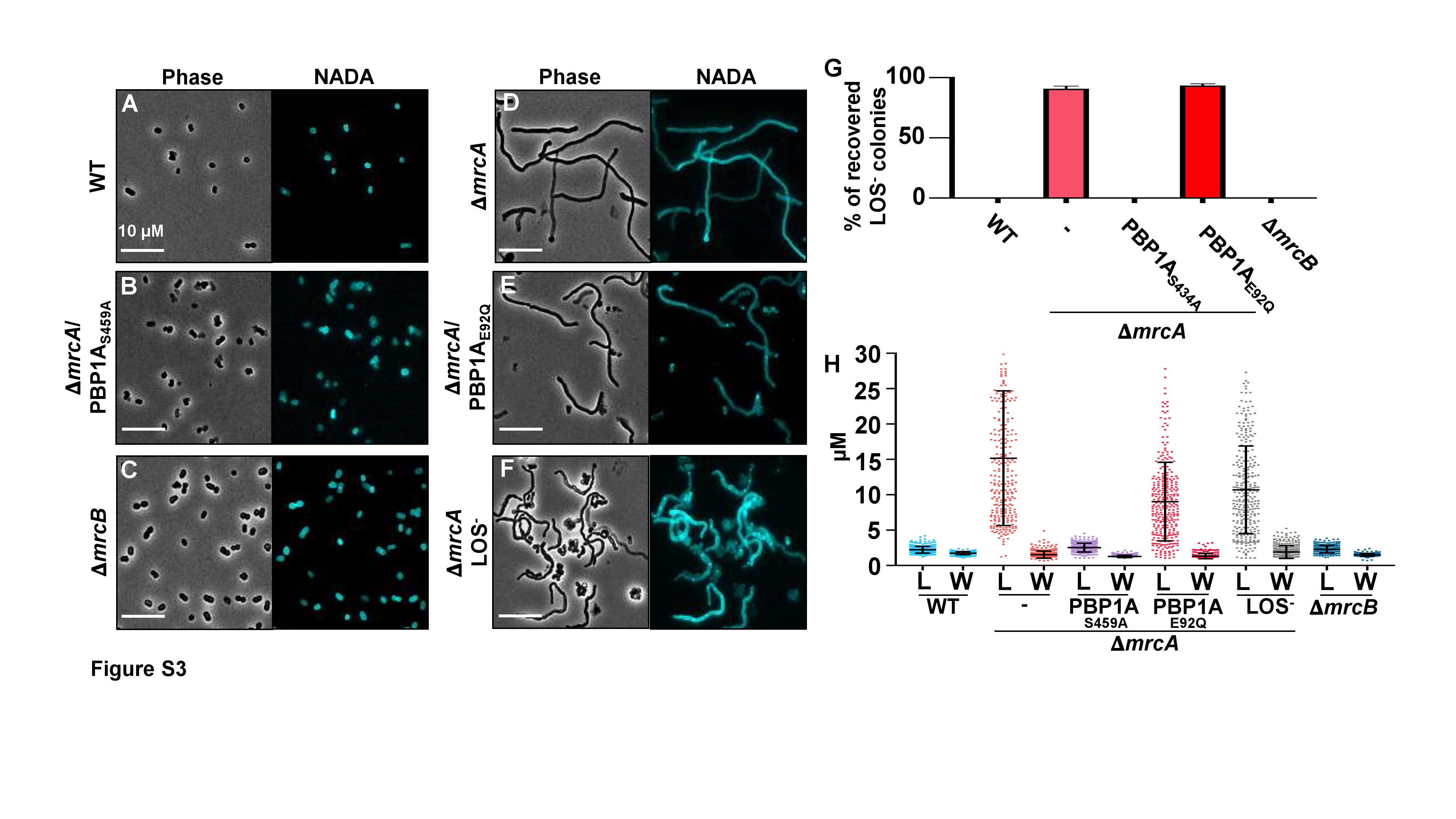

Supplement: FIG S3 [file mbio.02185-20-sf003.tif]

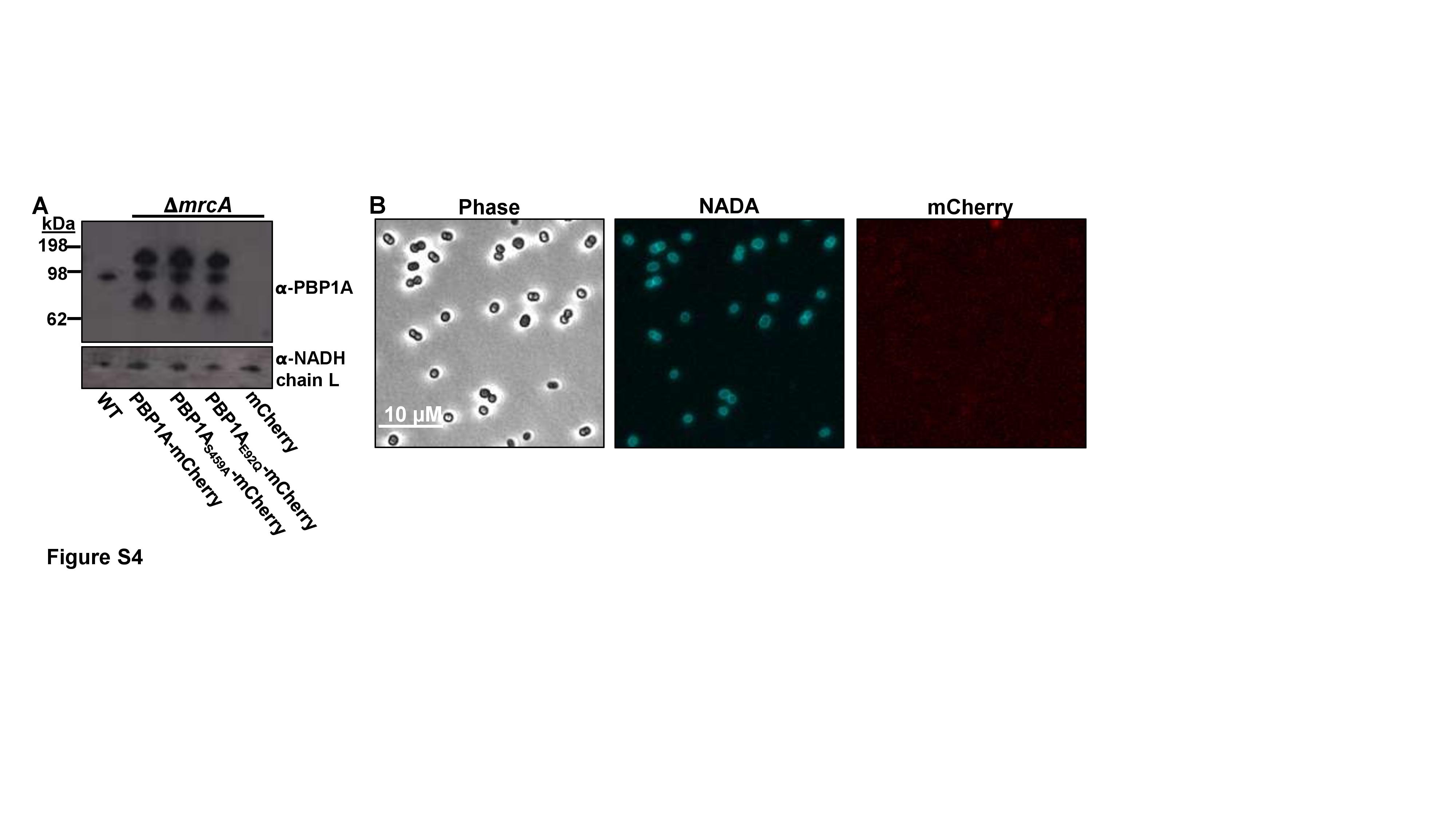

Supplement: FIG S4 [file mbio.02185-20-sf004.tif]

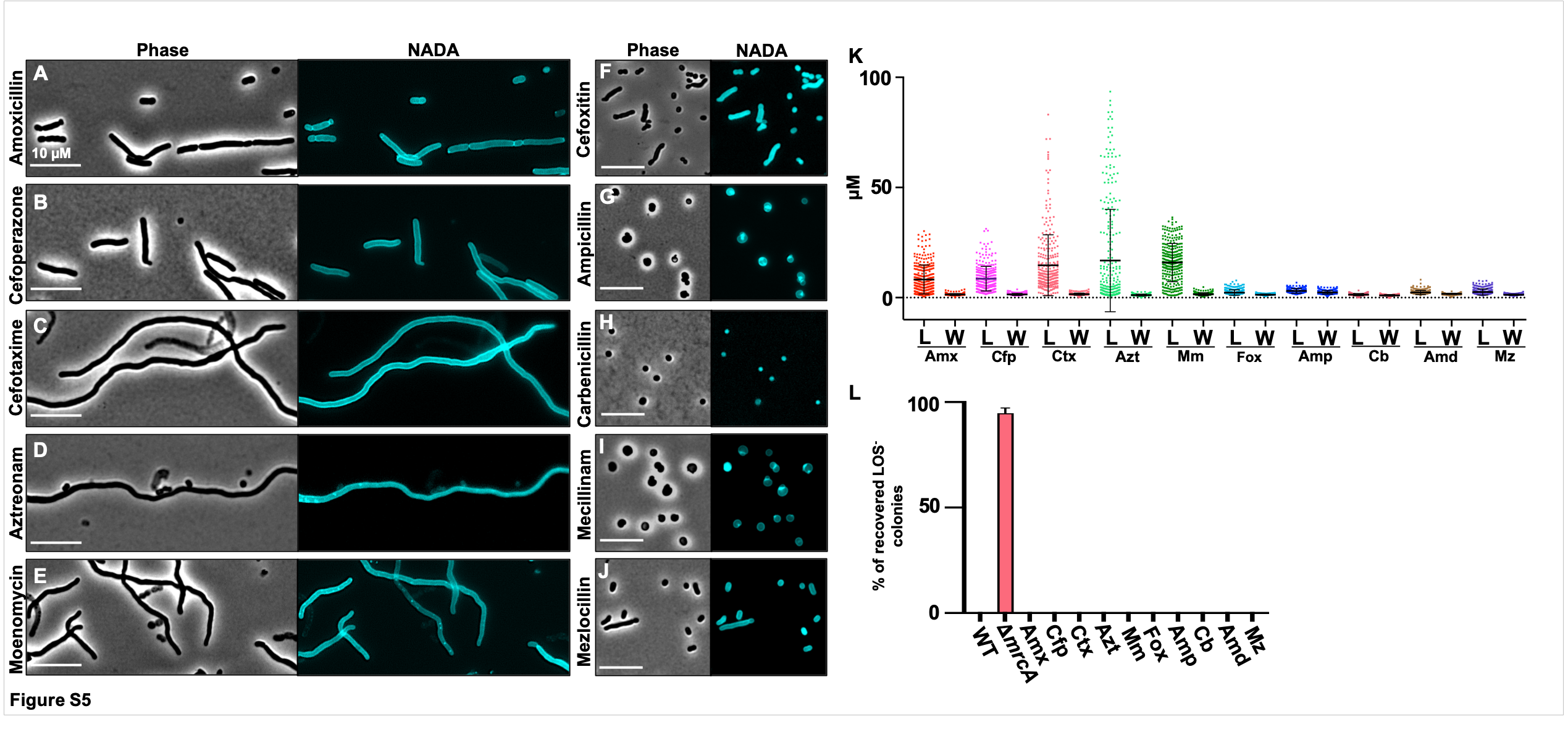

Supplement: FIG S5 [file mbio.02185-20-sf005.tif]

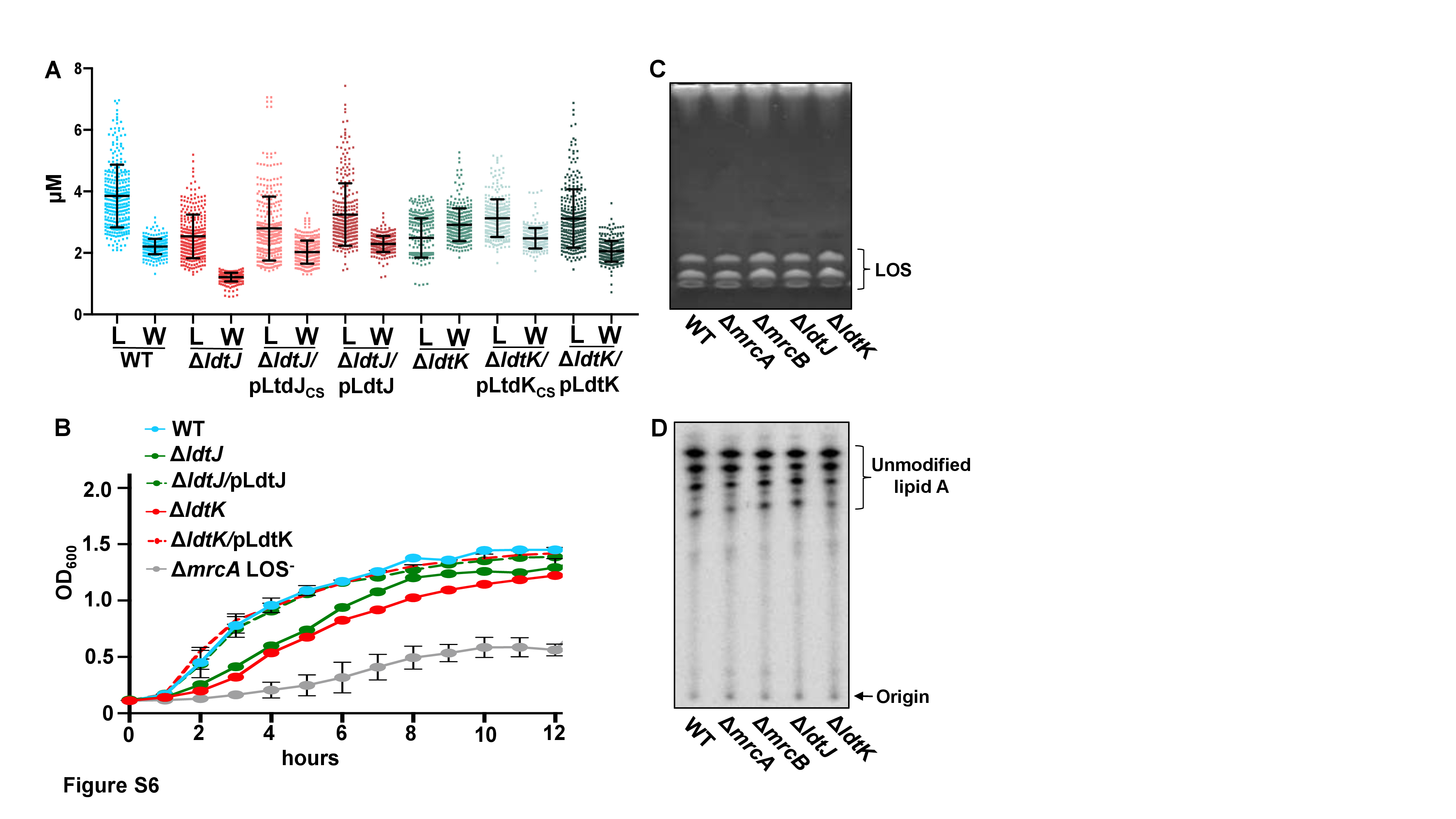

Supplement: FIG S6 [file mbio.02185-20-sf006.tif]
