# Supplementary material for: Septal Class A Penicillin-Binding Protein Activity and ld-Transpeptidases Mediate Selection of Colistin-Resistant Lipooligosaccharide-Deficient Acinetobacter baumannii
Source: mBio. 2021 Jan 5;12(1):e02185-20. doi: 10.1128/mBio.02185-20 (PMC8545086; doi:10.1128/mBio.02185-20)
Supplement: TABLE S1 [file mbio.02185-20-st001.docx]

| **Table S1A: Muropeptide composition of wild type and mutant *A. baumannii* strain ATCC 17978** | | | | | | | | | | | | | | | | | | | | | | | | | | | | | | |
| --- | --- | --- | --- | --- | --- | --- | --- | --- | --- | --- | --- | --- | --- | --- | --- | --- | --- | --- | --- | --- | --- | --- | --- | --- | --- | --- | --- | --- | --- | --- |
|  |  | | **Relative % of Each Muropeptide** | | | | | | | | | | | | | | | | | | | | | | | | | | | |
|  |  | | **WT Logarithmic** | **WT Stationary** | | | **Δ*mrcA* Logarithmic** | | | **Δ*mrcA* Stationary** | | | **Δ*mrcA* LOS^-^ Logarithmic** | | | **Δ*mrcA* LOS^-^ Stationary** | | | **Δ*ldtJ* Logarithmic** | | | **Δ*ldtJ* Stationary** | | | **Δ*ldtK* Logarithmic** | | | **Δ*ldtK* Stationary** | | |
| **Peak #** | **Name** | | **Peak Mean**  **±Variation** | **Peak Mean**  **±Variation** | | | **Peak Mean**  **±Variation** | | | **Peak Mean**  **±Variation** | | | **Peak Mean**  **±Variation** | | | **Peak Mean**  **±Variation** | | | **Peak Mean**  **±Variation** | | | **Peak Mean**  **±Variation** | | | **Peak Mean**  **±Variation** | | | **Peak Mean**  **±Variation** | | |
| **1** | **Tri** | | **2.9 ±0.13** | **4.9 ±0.2** | | | **3.9 ±0.3** | | | **7.7 ±1.24** | | | **2.3 ±0.04** | | | **3.3 ±0.02** | | | **0.0 ±0** | | | **1.2 ±0.08** | | | **2.9 ±0** | | | **5.1 ±0.41** | | |
| **2** | **Tri-D-Asn** | | **0.9 ±0.34** | **0.1 ±0.21** | | | **0.7 ±0.01** | | | **0.0 ±0** | | | **0.0 ±0** | | | **0.0 ±0** | | | **0.0 ±0** | | | **0.0 ±0** | | | **1.4 ±0.01** | | | **1.0 ±0.04** | | |
| **3** | **Tri-D-Lys** | | **0.2 ±0.36** | **6.7 ±0.53** | | | **0.5 ±0.1** | | | **6.0 ±0.91** | | | **0.0 ±0** | | | **0.1 ±0.2** | | | **0.0 ±0** | | | **0.0 ±0** | | | **0.0 ±0** | | | **3.8 ±0.72** | | |
| **4** | **TetraGly4** | | **2.1 ±0.12** | **0.2 ±0.37** | | | **2.1 ±0.04** | | | **0.0 ±0** | | | **0.6 ±0.26** | | | **0.1 ±0.2** | | | **0.0 ±0** | | | **0.0 ±0** | | | **3.4 ±0.33** | | | **2.3 ±0.24** | | |
| **5** | **Tetra-D-Lys** | | **0.0 ±0** | **3.9 ±1.24** | | | **0.0 ±0** | | | **6.9 ±2.94** | | | **0.0 ±0** | | | **0.0 ±0** | | | **0.0 ±0** | | | **0.0 ±0** | | | **0.0 ±0** | | | **2. ±2.07** | | |
| **6** | **Tetra** | | **21.1 ±1.06** | **10.3 ±1.23** | | | **21.6 ±0.54** | | | **10.5 ±2.32** | | | **25.7 ±1.14** | | | **22.3 ±1.34** | | | **30.1 ±0.08** | | | **30.0 ±0.91** | | | **26.9 ±0.87** | | | **18.6 ±1.79** | | |
| **7** | **Tetra-D-Arg** | | **0.0 ±0** | **0.3 ±0.21** | | | **0.1 ±0.24** | | | **0.1 ±0.24** | | | **0.9 ±0.19** | | | **0.5 ±0.15** | | | **0.0 ±0** | | | **0.7 ±0.34** | | | **0.1 ±0.21** | | | **0.4 ±0.18** | | |
| **8** | **TetraTriDapGly4** | | **0.0 ±0** | **0.3 ±0.19** | | | **0.5 ±0.07** | | | **0.0 ±0** | | | **0.0 ±0** | | | **0.0 ±0** | | | **0.0 ±0** | | | **0.0 ±0** | | | **1.1 ±0.14** | | | **1.9 ±0.07** | | |
| **9** | **TriTri(Dap)/TriTriDap-D-Lys** | | **0.4 ±0.16** | **2.6 ±0.11** | | | **1.1 ±0.32** | | | **2.5 ±0.04** | | | **0.5 ±0.19** | | | **0.4 ±0.25** | | | **0.0 ±0** | | | **0.0 ±0** | | | **0.9 ±0.28** | | | **3.3 ±0.28** | | |
| **10** | **TetraTri(Dap)/TriTriDap-D-Arg** | | **0.3 ±0.49** | **2.9 ±1.94** | | | **0.6 ±1.18** | | | **2.5 ±2.4** | | | **0.0 ±0** | | | **0.0 ±0** | | | **0.0 ±0** | | | **0.0 ±0** | | | **0.4 ±0.74** | | | **4.3 ±2.85** | | |
| **11** | **TetraTri** | | **3.7 ±0.91** | **3.7 ±1.87** | | | **6.1 ±1.3** | | | **4.3 ±2.74** | | | **4.4 ±0.08** | | | **2.8 ±0.1** | | | **0.2 ±0.31** | | | **0.0 ±0** | | | **12.4 ±1.68** | | | **10.6 ±2.02** | | |
| **12** | **TetraTri-D-Lys** | | **0.4 ±0.19** | **2.2 ±1.6** | | | **0.5 ±0.08** | | | **1.1 ±0.79** | | | **0.3 ±0.03** | | | **1.8 ±0.3** | | | **0.0 ±0** | | | **0.2 ±0.38** | | | **1.9 ±0.78** | | | **1.9 ±0.22** | | |
| **13** | **TetraTri-D-Lys** | | **0.3 ±0.1** | **3.3 ±1.68** | | | **0.6 ±0.08** | | | **2.4 ±0.85** | | | **0.4 ±0.75** | | | **1.8 ±0.73** | | | **0.0 ±0** | | | **0.0 ±0** | | | **0.2 ±0.35** | | | **1.0 ±0** | | |
| **14** | **TetraTri-D-Arg** | | **0.2 ±0.48** | **6.7 ±1.16** | | | **0.8 ±0.42** | | | **7.5 ±2.76** | | | **0.9 ±0.31** | | | **0.6 ±0.34** | | | **0.7 ±0.39** | | | **2.9 ±0.72** | | | **0.0 ±0** | | | **3.2 ±1.52** | | |
| **15** | **TetraTetra** | | **35.9 ±0.6** | **16.0 ±0.66** | | | **35.1 ±0.59** | | | **17.9 ±4.36** | | | **36.8 ±0.79** | | | **35.3 ±0.46** | | | **38.2 ±0** | | | **29.9 ±2.09** | | | **30.7 ±1.41** | | | **15.8 ±0.14** | | |
| **16** | **TetraTetraTri/TetraTetraTriDap** | | **0.9 ±0.2** | **1.6 ±0.41** | | | **1.3 ±0.32** | | | **1.6 ±0.59** | | | **1.0 ±0.24** | | | **0.8 ±0.22** | | | **0.0 ±0** | | | **0.0 ±0** | | | **1.7 ±0.17** | | | **2.0 ±0.26** | | |
| **17** | **TetraTetraTri/TetraTetraTriDap** | | **0.1 ±0.2** | **1.2 ±0.23** | | | **0.7 ±0.77** | | | **1.2 ±1.12** | | | **0.3 ±0.02** | | | **0.6 ±0.1** | | | **0.0 ±0** | | | **0.0 ±0** | | | **0.3 ±0.09** | | | **0.9 ±0.73** | | |
| **18** | **TriTriDap-D-Met** | | **0.6 ±0.35** | **1.6 ±0.85** | | | **0.3 ±0.64** | | | **0.8 ±0.8** | | | **0.6 ±0.13** | | | **0.6 ±0.04** | | | **0.0 ±0** | | | **0.3 ±0.54** | | | **1.0 ±0.27** | | | **1.2 ±0.74** | | |
| **19** | **TetraTetraTetra** | | **18.5 ±1.04** | **11.0 ±0.46** | | | **11.1 ±0.16** | | | **6.7 ±3.86** | | | **11.8 ±0.45** | | | **13.1 ±0.13** | | | **18.3 ±0.18** | | | **14.5 ±0.64** | | | **7.1 ±0.04** | | | **3.4 ±0.65** | | |
| **20** | **TetraTri-D-Met** | | **0.0 ±0** | **1.8 ±0.4** | | | **0.0 ±0** | | | **1.0 ±0.29** | | | **0.2 ±0.32** | | | **0.1 ±0.18** | | | **0.0 ±0** | | | **0.6 ±0.44** | | | **0.0 ±0** | | | **0.9 ±0.29** | | |
| **21** | **TetraTri Anh/TetraTetraTetraTri** | | **4.7 ±0.25** | **2.6 ±0.3** | | | **1.7 ±0.81** | | | **1.3 ±0.43** | | | **1.9 ±0.94** | | | **2.3 ±0.24** | | | **4.3 ±0.08** | | | **3.2 ±0.06** | | | **1.0 ±0.58** | | | **0.5 ±0.39** | | |
| **22** | **TetraTetra Anh I** | | **1.1 ±0.11** | **1.2 ±0.08** | | | **1.9 ±0.27** | | | **1.3 ±0.14** | | | **2.3 ±0.2** | | | **2.7 ±0.48** | | | **2.2 ±0.68** | | | **2.0 ±0.94** | | | **0.8 ±0.03** | | | **0.7 ±0.15** | | |
| **23** | **TetraTetra Anh II** | | **0.9 ±0.12** | **1.2 ±0.32** | | | **1.7 ±0.1** | | | **2.4 ±0.44** | | | **1.6 ±0.04** | | | **2.1 ±0.41** | | | **1.1 ±0.2** | | | **1.5 ±0.43** | | | **0.6 ±0.12** | | | **0.9 ±0.13** | | |
| **24** | **TetraTetraTetra Anh** | | **2.1 ±0.24** | **1.6 ±0.1** | | | **2.4 ±0.47** | | | **2.8 ±0.82** | | | **2.2 ±0.28** | | | **3.3 ±0.23** | | | **3.0 ±0.46** | | | **2.7 ±0.35** | | | **0.7 ±0.14** | | | **0.6 ±0.13** | | |
| **Sum of known peaks** | | | **96.9 ±1.52** | **87.8 ±3.23** | | | **95.2 ±0.46** | | | **88.4 ±5.44** | | | **94.6 ±0.93** | | | **94.5 ±1.33** | | | **97.7 ±0.54** | | | **89.4 ±0.57** | | | **95.1 ±2.13** | | | **86.6 ±1.08** | | |
|  |  | |  |  | | |  | | |  | | |  | | |  | | |  | | |  | | |  | | |  | | |
| **Monomers (Total)** | | | **27.8 ±1.56** | **30.2 ±0.3** | | | **30.3 ±1.33** | | | **35.4 ±5.58** | | | **31.2 ±0.46** | | | **27.8 ±1.27** | | | **30.8 ±0.08** | | | **35.6 ±0.95** | | | **36.4 ±0.67** | | | **38.5 ±0.7** | | |
| **Monomers with modification** | | | **3.3 ±0.05** | **12.8 ±1.96** | | | **3.6 ±0.32** | | | **14.8 ±5.53** | | | **1.6 ±0.49** | | | **0.7 ±0.25** | | | **0.0 ±0** | | | **0.7 ±0.37** | | | **5.2 ±0.46** | | | **11.3 ±2.64** | | |
| **Monomer tri** | | | **2.8 ±0.17** | **5.6 ±0.43** | | | **4.1 ±0.33** | | | **8.7 ±1.94** | | | **2.4 ±0.01** | | | **3.5 ±0.07** | | | **0.0 ±0** | | | **1.3 ±0.09** | | | **3.0 ±0.06** | | | **5.8 ±0.4** | | |
| **Monomer tri-D-Asn** | | | **1.0 ±0.33** | **0.1 ±0.23** | | | **0.8 ±0** | | | **0.0 ±0** | | | **0.0 ±0** | | | **0.0 ±0** | | | **0.0 ±0** | | | **0.0 ±0** | | | **1.5 ±0.02** | | | **1.2 ±0.03** | | |
| **Monomer tri-D-Lys** | | | **0.2 ±0.37** | **7.7 ±0.32** | | | **0.5 ±0.1** | | | **6.8 ±1.44** | | | **0.0 ±0** | | | **0.1 ±0.21** | | | **0.0 ±0** | | | **0.0 ±0** | | | **0.0 ±0** | | | **4.4 ±0.77** | | |
| **Monomer tetraGly4** | | | **2.2 ±0.08** | **0.2 ±0.41** | | | **2.2 ±0.03** | | | **0.0 ±0** | | | **0.6 ±0.28** | | | **0.1 ±0.21** | | | **0.0 ±0** | | | **0.0 ±0** | | | **3.6 ±0.26** | | | **2.7 ±0.31** | | |
| **Monomer tetra-D-Lys** | | | **0.0 ±0** | **4.5 ±1.24** | | | **0.0 ±0** | | | **7.9 ±3.81** | | | **0.0 ±0** | | | **0.0 ±0** | | | **0.0 ±0** | | | **0.0 ±0** | | | **0.0 ±0** | | | **2.6 ±2.35** | | |
| **Monomer tetra** | | | **21.8 ±1.43** | **11.8 ±1.83** | | | **22.7 ±0.67** | | | **11.9 ±1.89** | | | **27.2 ±0.93** | | | **23.6 ±1.08** | | | **30.8 ±0.08** | | | **33.5 ±1.23** | | | **28.2 ±0.27** | | | **21.5 ±2.33** | | |
| **Monomer tetra-D-Arg** | | | **0.0 ±0** | **0.3 ±0.25** | | | **0.1 ±0.25** | | | **0.1 ±0.28** | | | **1.0 ±0.21** | | | **0.5 ±0.16** | | | **0.0 ±0** | | | **0.7 ±0.37** | | | **0.1 ±0.21** | | | **0.4 ±0.21** | | |
|  |  | |  |  | | |  | | |  | | |  | | |  | | |  | | |  | | |  | | |  | | |
| **Dimers (Total)** | | | **49.9 ±0.59** | **52.4 ±0.42** | | | **53.5 ±0.05** | | | **50.8 ±0.79** | | | **52.61 ±0.74** | | | **53.42 ±0.56** | | | **47.6 ±0.85** | | | **45.3 ±1.41** | | | **53.4 ±0.43** | | | **53.5 ±1.43** | | |
| **Dimers with modification** | | | **2.0 ±1.28** | **21.1 ±1.48** | | | **4.0 ±0.55** | | | **17.3 ±4.87** | | | **2.96 ±1.12** | | | **5.63 ±0.3** | | | **0.7 ±0.39** | | | **4.4 ±0.16** | | | **5.2 ±1.29** | | | **15.5 ±1.59** | | |
| **Dimer anhydrous** | | | **6.8 ±0.14** | **5.6 ±0.28** | | | **5.5 ±1.21** | | | **5.7 ±0.47** | | | **6.13 ±0.88** | | | **7.47 ±0.28** | | | **7.7 ±0.35** | | | **7.5 ±0.54** | | | **2.5 ±0.58** | | | **2.5 ±0.5** | | |
|  |  | |  |  | | |  | | |  | | |  | | |  | | |  | | |  | | |  | | |  | | |
| **Trimers (Total)** | | | **22.3 ±0.96** | **17.4 ±0.72** | | | **16.2 ±1.39** | | | **13.8 ±6.38** | | | **16.19 ±1.2** | | | **18.77 ±0.7** | | | **21.7 ±0.77** | | | **19.1 ±0.46** | | | **10.2 ±0.23** | | | **7.9 ±2.14** | | |
| **Trimer anhydrous** | | | **2.2 ±0.21** | **1.8 ±0.04** | | | **2.5 ±0.48** | | | **3.2 ±0.73** | | | **2.36 ±0.31** | | | **3.46 ±0.29** | | | **3.0 ±0.48** | | | **3.0 ±0.37** | | | **0.7 ±0.16** | | | **0.7 ±0.15** | | |
|  |  | |  |  | | |  | | |  | | |  | | |  | | |  | | |  | | |  | | |  | | |
| **Tripeptides (Total)** | | | **10.0 ±0.87** | **30.9 ±1.09** | | | **12.3 ±0.24** | | | **30.3 ±5.57** | | | **8.00 ±0.06** | | | **9.90 ±0.27** | | | **2.6 ±0.29** | | | **5.5 ±0.14** | | | **14.9 ±0.76** | | | **28.7 ±2.13** | | |
| **Tripeptides with modifications** | | | **2.7 ±0.86** | **20.7 ±1.75** | | | **4.0 ±0.55** | | | **17.3 ±4.47** | | | **2.05 ±0.38** | | | **3.46 ±0.1** | | | **0.3 ±0.19** | | | **2.3 ±0.22** | | | **5.0 ±0.98** | | | **15.9 ±1.83** | | |
| **Tetrapeptides (Total)** | | | **89.8 ±0.95** | **67.4 ±1.2** | | | **86.8 ±0.04** | | | **68.4 ±5.68** | | | **91.74 ±0.17** | | | **89.87 ±0.4** | | | **97.4 ±0.29** | | | **94.5 ±0.14** | | | **84.1 ±1** | | | **68.3 ±1.98** | | |
| **Tetrapeptides with modifications** | | | **2.6 ±0.47** | **13.2 ±1.69** | | | **3.6 ±0.32** | | | **14.8 ±5.94** | | | **2.52 ±0.24** | | | **2.88 ±0.05** | | | **0.3 ±0.19** | | | **2.8 ±0.01** | | | **5.4 ±0.14** | | | **10.9 ±2.39** | | |
|  |  | |  |  | | |  | | |  | | |  | | |  | | |  | | |  | | |  | | |  | | |
| **3-3 Crosslinks** | | | **1.0 ±0** | **5.3 ±1.8** | | | **2.0 ±0.37** | | | **4.3 ±1.45** | | | **1.04 ±0.27** | | | **1.03 ±0.28** | | | **0.0 ±0** | | | **0.2 ±0.3** | | | **2.4 ±0.77** | | | **7.3 ±1.48** | | |
| **Chain ends (anhydrous)** | | | **4.1 ±0.14** | **3.4 ±0.12** | | | **3.6 ±0.76** | | | **3.9 ±0.48** | | | **3.85 ±0.54** | | | **4.89 ±0.23** | | | **4.9 ±0.01** | | | **4.8 ±0.15** | | | **1.5 ±0.34** | | | **1.5 ±0.3** | | |
|  |  | |  |  | | |  | |  |  | | |  | | |  | | |  | | |  | | |  | | |  | | |
| **Degree of crosslinks** | | | **39.1 ±0.94** | **37.8 ±0.27** | | | **37.5 ±0.9** | | | **34.6 ±3.85** | | | **37.10 ±0.43** | | | **39.22 ±0.75** | | | **38.2 ±0.08** | | | **35.4 ±0.39** | | | **33.5 ±0.37** | | | **32.1 ±0.7** | | |
| **% peptides in cross-links** | | | **72.2 ±1.56** | **69.8 ±0.3** | | | **69.6 ±1.33** | | | **64.6 ±5.58** | | | **68.80 ±0.46** | | | **72.19 ±1.27** | | | **69.3 ±0.08** | | | **64.4 ±0.95** | | | **63.6 ±0.67** | | | **61.5 ±0.7** | | |
| **^a^denotes variation of two replicates** | | | |  | |  |  | |  |  | |  |  | |  |  | |  |  | |  |  | |  |  | |  |  | |  |
| **Table S1B: Muropeptide composition of wild type and mutant *A. baumannii* strain ATCC 19606** | | | | | | | | | | | | | | | | | | | | | | | | | | | | |  |  |
|  | |  | | | **Relative % of Each Muropeptide** | | | | | | | | | | | | | | | | | | | | | | | |  |  |
|  | |  | | | **WT Logarithmic** | | | **WT Stationary** | | | **LOS^-^ Logarithmic** | | | **LOS^-^ Stationary** | | | **Δ*ldtJ* Logarithmic** | | | **Δ*ldtJ* Stationary** | | | **Δ*ldtK* Logarithmic** | | | **Δ*ldtK* Stationary** | | |  |  |
| **Peak number** | | **Name** | | | **Peak Mean ±Variation^a^** | | | **Peak Mean ±Variation** | | | **Peak Mean**  **±Variation** | | | **Peak Mean**  **±Variation** | | | **Peak Mean**  **±Variation** | | | **Peak Mean**  **±Variation** | | | **Peak Mean**  **±Variation** | | | **Peak Mean**  **±Variation** | | |  |  |
| **1** | | **Tri** | | | 2.0 ±0.3 | | | 2.6 ±0.94 | | | 2.0 ±0.75 | | | 2.7 ±0.11 | | | 0.0 ±0 | | | 0.3 ±0.03 | | | 1.5 ±0.38 | | | 2.5 ±0.02 | | |  |  |
| **2** | | **Tri-D-Asn** | | | 0.5 ±0.01 | | | 0.0 ±0 | | | 0.0 ±0 | | | 0.0 ±0 | | | 0.0 ±0 | | | 0.0 ±0 | | | 0.6 ±1.22 | | | 0.5 ±0.04 | | |  |  |
| **3** | | **Tri-D-Lys** | | | 1.2 ±2.46 | | | 1.5 ±2.93 | | | 0.0 ±0 | | | 0.0 ±0 | | | 0.0 ±0 | | | 0.0 ±0 | | | 1.4 ±2.7 | | | 0.0 ±0 | | |  |  |
| **4** | | **TetraGly4** | | | 1.0 ±2.01 | | | 1.4 ±2.8 | | | 0.0 ±0 | | | 1.1 ±0.07 | | | 0.0 ±0 | | | 0.5 ±0.02 | | | 1.4 ±2.76 | | | 3.0 ±0.34 | | |  |  |
| **5** | | **Tetra-D-Lys** | | | 0.0 ±0 | | | 0.0 ±0 | | | 0.0 ±0 | | | 0.0 ±0 | | | 0.0 ±0 | | | 0.0 ±0 | | | 0.0 ±0 | | | 0.0 ±0 | | |  |  |
| **6** | | **Tetra** | | | 20.2 ±0.32 | | | 15.0 ±1.32 | | | 26.4 ±0.66 | | | 20.3 ±1.11 | | | 31.7 ±0.16 | | | 22.8 ±0.51 | | | 19.2 ±1.4 | | | 19.5±1.47 | | |  |  |
| **7** | | **Tetra-D-Arg** | | | 0.0 ±0 | | | 0.1 ±0.15 | | | 0.4 ±0.86 | | | 0.0 ±0 | | | 0.0 ±0 | | | 0.1 ±0.17 | | | 0.2 ±0.44 | | | 0.2±0.46 | | |  |  |
| **8** | | **TetraTriDapGly4** | | | 0.4 ±0.05 | | | 0.7 ±0.15 | | | 0.0 ±0 | | | 0.2 ±0.32 | | | 0.0 ±0 | | | 0.0 ±0 | | | 1.9 ±0 | | | 2.6±0.37 | | |  |  |
| **9** | | **TriTri(Dap)/TriTriDap-D-Lys** | | | 0.5 ±0.19 | | | 0.8 ±0.55 | | | 0.3 ±0.1 | | | 0.8 ±0.42 | | | 0.0 ±0 | | | 0.0 ±0 | | | 0.8 ±0.31 | | | 1.1 ±0.38 | | |  |  |
| **10** | | **TetraTri(Dap)/TriTriDap-D-Arg** | | | 0.0 ±0 | | | 0.1 ±0.25 | | | 0.0 ±0 | | | 0.0 ±0 | | | 0.0 ±0 | | | 0.0 ±0 | | | 0.1 ±0.23 | | | 0.5 ±0.91 | | |  |  |
| **11** | | **TetraTri** | | | 5.6 ±0.22 | | | 4.6 ±0.81 | | | 0.0 ±0 | | | 4.2 ±0.8 | | | 0.1 ±0.29 | | | 0.1 ±0.24 | | | 16.2 ±4.89 | | | 15.8 ±1.23 | | |  |  |
| **12** | | **TetraTri-D-Lys** | | | 0.5 ±0.12 | | | 1.6 ±0.18 | | | 2.5 ±2.79 | | | 0.7 ±0.94 | | | 0.0 ±0 | | | 0.5 ±0.14 | | | 2.2 ±1.64 | | | 2.3 ±0.94 | | |  |  |
| **13** | | **TetraTri-D-Lys** | | | 0.3 ±0.06 | | | 0.4 ±0.8 | | | 0.4 ±0.8 | | | 1.1 ±0.21 | | | 0.0 ±0 | | | 0.0 ±0 | | | 0.1 ±0.26 | | | 0.2 ±0.41 | | |  |  |
| **14** | | **TetraTri-D-Arg** | | | 0.0 ±0 | | | 1.8 ±0.78 | | | 0.2 ±0.36 | | | 1.1 ±0.55 | | | 0.0 ±0 | | | 2.2 ±0.71 | | | 1.0±1.03 | | | 1.4 ±1.28 | | |  |  |
| **15** | | **TetraTetra** | | | 35.4 ±2.13 | | | 30.7 ±3.6 | | | 35.7 ±2.97 | | | 31.4 ±0.82 | | | 35.1 ±1.82 | | | 31.3 ±0.28 | | | 26.5 ±2.31 | | | 23.2 ±0.73 | | |  |  |
| **16** | | **TetraTetraTri/TetraTetraTriDap** | | | 1.3 ±0.14 | | | 2.4 ±0.65 | | | 0.3 ±0.67 | | | 1.6 ±0.18 | | | 0.0 ±0 | | | 0.1 ±0.16 | | | 2.8 ±0.33 | | | 2.7 ±0.5 | | |  |  |
| **17** | | **TetraTetraTri/TetraTetraTriDap** | | | 0.6 ±0.79 | | | 0.4 ±0.84 | | | 0.5 ±0.15 | | | 0.7 ±0.24 | | | 0.0 ±0 | | | 0.3 ±0.31 | | | 0.9 ±1.45 | | | 0.6 ±0.8 | | |  |  |
| **18** | | **TriTriDap-D-Met** | | | 0.4 ±0.75 | | | 0.4 ±0.22 | | | 0.3 ±0.51 | | | 0.4 ±0.84 | | | 0.0 ±0 | | | 0.3 ±0.69 | | | 0.7 ±0.77 | | | 0.6 ±0.76 | | |  |  |
| **19** | | **TetraTetraTetra** | | | 14.3 ±1.47 | | | 14.5 ±1.15 | | | 11.7 ±0.94 | | | 11.7 ±0.8 | | | 14.1 ±0.95 | | | 13.9 ±0.09 | | | 5.2 ±1.29 | | | 3.6 ±0.18 | | |  |  |
| **20** | | **TetraTri-D-Met** | | | 0.4 ±0.76 | | | 0.4 ±0.76 | | | 0.1 ±0.24 | | | 0.2 ±0.43 | | | 0.0 ±0 | | | 0.0 ±0 | | | 0.3 ±0.57 | | | 0.0 ±0 | | |  |  |
| **21** | | **TetraTri Anh/TetraTetraTetraTri** | | | 3.2 ±0.68 | | | 3.7 ±0.14 | | | 2.0 ±0.49 | | | 2.3 ±0.06 | | | 2.7 ±0.39 | | | 3.5 ±0.17 | | | 1.0±1.22 | | | 0.8 ±0.61 | | |  |  |
| **22** | | **TetraTetra Anh I** | | | 2.5 ±0.25 | | | 2.0 ±0.9 | | | 2.7 ±0.14 | | | 2.2 ±0.91 | | | 3.5 ±0.08 | | | 3.0 ±0.22 | | | 1.6±0.16 | | | 1.9 ±0.16 | | |  |  |
| **23** | | **TetraTetra Anh II** | | | 1.2 ±0.22 | | | 1.2 ±0.18 | | | 1.9 ±0.3 | | | 2.7 ±0.16 | | | 2.4 ±0.8 | | | 2.5 ±0.63 | | | 1.5±0.93 | | | 2.4 ±0.06 | | |  |  |
| **24** | | **TetraTetraTetra Anh** | | | 3.0 ±0.8 | | | 3.3 ±0.32 | | | 3.6 ±0.36 | | | 4.7 ±0.29 | | | 4.8 ±0.81 | | | 6.2 ±0.22 | | | 1.3±0.4 | | | 1.6 ±0.48 | | |  |  |
| **Sum of known peaks** | | | | | 94.6 ±1.59 | | | 89.5 ±3.88 | | | 90.8 ±6.75 | | | 90.2 ±3.97 | | | 94.6 ±1.14 | | | 87.7 ±0 | | | 88.3 ±1.19 | | | 87.1 ±1.3 | | |  |  |
|  | |  | | |  | | |  | | |  | | |  | | |  | | |  | | |  | | |  | | |  |  |
| **Monomers (Total)** | | | | | 26.4 ±0.69 | | | 22.9 ±0.87 | | | 31.7 ±2.96 | | | 26.8 ±2.19 | | | 33.6 ±0.22 | | | 26.9 ±0.76 | | | 27.4 ±0.57 | | | 29.6 ±0.24 | | |  |  |
| **Monomers with modification** | | | | | 2.9 ±0.43 | | | 3.3 ±0.45 | | | 0.5 ±0.91 | | | 1.3 ±0.03 | | | 0.0 ±0 | | | 0.6 ±0.21 | | | 4.0 ±0.89 | | | 4.3 ±1.02 | | |  |  |
| **Monomer tri** | | | | | 2.1 ±0.28 | | | 2.9 ±1.17 | | | 2.2 ±0.98 | | | 3.0 ±0 | | | 0.0 ±0 | | | 0.3 ±0.03 | | | 1.7 ±0.4 | | | 2.9 ±0.07 | | |  |  |
| **Monomer tri-D-Asn** | | | | | 0.6 ±0 | | | 0.0 ±0 | | | 0.0 ±0 | | | 0.0 ±0 | | | 0.0 ±0 | | | 0.0 ±0 | | | 0.7 ±1.37 | | | 0.5 ±0.05 | | |  |  |
| **Monomer tri-D-Lys** | | | | | 1.3 ±2.57 | | | 1.7 ±3.34 | | | 0.0 ±0 | | | 0.0 ±0 | | | 0.0 ±0 | | | 0.0 ±0 | | | 1.5 ±3.07 | | | 0.0 ±0 | | |  |  |
| **Monomer tetraGly4** | | | | | 1.1 ±2.14 | | | 1.5 ±3.06 | | | 0.0 ±0 | | | 1.3 ±0.03 | | | 0.0 ±0 | | | 0.5 ±0.02 | | | 1.6 ±3.1 | | | 3.5 ±0.44 | | |  |  |
| **Monomer tetra-D-Lys** | | | | | 0.0 ±0 | | | 0.0 ±0 | | | 0.0 ±0 | | | 0.0 ±0 | | | 0.0 ±0 | | | 0.0 ±0 | | | 0.0 ±0 | | | 0.0 ±0 | | |  |  |
| **Monomer tetra** | | | | | 21.4 ±0.02 | | | 16.7 ±0.75 | | | 29.1 ±2.89 | | | 22.6 ±2.22 | | | 33.6 ±0.22 | | | 26.0 ±0.58 | | | 21.7 ±1.87 | | | 22.4 ±1.35 | | |  |  |
| **Monomer tetra-D-Arg** | | | | | 0.0 ±0 | | | 0.1 ±0.17 | | | 0.5 ±0.91 | | | 0.0 ±0 | | | 0.0 ±0 | | | 0.1 ±0.19 | | | 0.3 ±0.5 | | | 0.3 ±0.53 | | |  |  |
|  | |  | | |  | | |  | | |  | | |  | | |  | | |  | | |  | | |  | | |  |  |
| **Dimers (Total)** | | | | | 53.3 ±0.08 | | | 54.1 ±0.74 | | | 50.5 ±3.06 | | | 52.4 ±2.61 | | | 46.5 ±0.31 | | | 49.6 ±0.95 | | | 61.0 ±2.45 | | | 60.6 ±1.19 | | |  |  |
| **Dimers with modification** | | | | | 2.6 ±0.49 | | | 6.8 ±1.31 | | | 4.1 ±3.97 | | | 4.9 ±2.01 | | | 0.0 ±0 | | | 3.5 ±0.13 | | | 7.9 ±0.41 | | | 9.5 ±1.86 | | |  |  |
| **Dimer anhydrous** | | | | | 7.3 ±1.33 | | | 7.7 ±1.69 | | | 7.2 ±1.25 | | | 8.0 ±0.54 | | | 9.2 ±1.46 | | | 10.2 ±0.77 | | | 4.6 ±0.44 | | | 5.9 ±0.86 | | |  |  |
|  | |  | | |  | | |  | | |  | | |  | | |  | | |  | | |  | | |  | | |  |  |
| **Trimers (Total)** | | | | | 20.3 ±0.61 | | | 23.1 ±0.13 | | | 17.7 ±0.09 | | | 20.8 ±0.41 | | | 20.0 ±0.09 | | | 23.5 ±0.19 | | | 11.5 ±3.02 | | | 9.8 ±0.95 | | |  |  |
| **Trimer anhydrous** | | | | | 3.1 ±0.89 | | | 3.7 ±0.51 | | | 4.0 ±0.69 | | | 5.3 ±0.55 | | | 5.1 ±0.91 | | | 7.1 ±0.25 | | | 1.5 ±0.43 | | | 1.9 ±0.52 | | |  |  |
|  | |  | | |  | | |  | | |  | | |  | | |  | | |  | | |  | | |  | | |  |  |
| **Tripeptides (Total)** | | | | | 10.6 ±2.37 | | | 13.5 ±4.91 | | | 5.7 ±1.08 | | | 10.0 ±1.53 | | | 1.5 ±0.07 | | | 4.5 ±0.31 | | | 18.4 ±3.19 | | | 18.1 ±1.15 | | |  |  |
| **Tripeptides with modifications** | | | | | 3.7 ±2.61 | | | 5.8 ±2.53 | | | 2.3 ±2.18 | | | 3.1 ±1.2 | | | 0.0 ±0 | | | 1.9 ±0.46 | | | 7.0 ±1.77 | | | 6.3 ±1.21 | | |  |  |
| **Tetrapeptides (Total)** | | | | | 88.9 ±2.24 | | | 85.7 ±4.55 | | | 94.1 ±1.02 | | | 89.4 ±1.45 | | | 98.5 ±0.07 | | | 95.5 ±0.31 | | | 80.1 ±3.03 | | | 79.8 ±1.18 | | |  |  |
| **Tetrapeptides with modifications** | | | | | 1.9 ±2.68 | | | 4.3 ±3.39 | | | 2.2 ±2.69 | | | 3.1 ±0.83 | | | 0.0 ±0 | | | 2.2 ±0.54 | | | 4.9 ±3.08 | | | 7.5 ±1.67 | | |  |  |
|  | |  | | |  | | |  | | |  | | |  | | |  | | |  | | |  | | |  | | |  |  |
| **3-3 Crosslinks** | | | | | 1.4 ±0.08 | | | 2.2 ±0.1 | | | 0.6 ±0.37 | | | 1.6 ±0.31 | | | 0.0 ±0 | | | 0.4 ±0.45 | | | 3.4 ±0.24 | | | 4.0 ±0.14 | | |  |  |
| **Chain ends (anhydrous)** | | | | | 4.7 ±0.96 | | | 5.1 ±1.02 | | | 4.9 ±0.85 | | | 5.7 ±0.08 | | | 6.3 ±1.03 | | | 7.5 ±0.3 | | | 2.8 ±0.36 | | | 3.6 ±0.6 | | |  |  |
|  | |  | | |  | | |  | | |  | | |  | | |  | | |  | | |  | | |  | | |  |  |
| **Degree of crosslinks** | | | | | 40.2 ±0.45 | | | 42.4 ±0.46 | | | 37.1 ±1.46 | | | 40.1 ±1.02 | | | 36.6 ±0.09 | | | 40.4 ±0.34 | | | 38.2 ±0.79 | | | 36.8 ±0.03 | | |  |  |
| **% peptides in cross-links** | | | | | 73.6 ±0.69 | | | 77.1 ±0.87 | | | 68.3 ±2.96 | | | 73.2 ±2.19 | | | 66.4 ±0.22 | | | 73.1 ±0.76 | | | 72.6 ±0.57 | | | 70.4 ±0.24 | | |  |  |
| **^a^denotes variation of two replicates** | | | | | | | |  |  |  |  |  |  |  |  |  |  |  |  |  |  |  |  |  |  |  |  |  |  |  |
