# Supplementary material for: Septal Class A Penicillin-Binding Protein Activity and ld-Transpeptidases Mediate Selection of Colistin-Resistant Lipooligosaccharide-Deficient Acinetobacter baumannii
Source: mBio. 2021 Jan 5;12(1):e02185-20. doi: 10.1128/mBio.02185-20 (PMC8545086; doi:10.1128/mBio.02185-20)
Supplement: TABLE S2 [file mbio.02185-20-st002.docx]

| **Table S2.** Reduced muropeptides from *Acinetobacter baumannii* 17978 ∆*ldtK* | | | | |  |
| --- | --- | --- | --- | --- | --- |
| No | Proposed structure | Theoretical neutral mass (Da) | Determined neutral mass (Da) | Remarks |  |
| 2 | Tri-D-Asn | 984.4135 | 984.1322 | MS/MS: loss of 132 (terminal D-Asn) | |
| 3 | Tri-D-Lys | 998.4656 | 998.2389 |  | |
| 4 | TetraGly4 | 927.3921 | 927.1631 |  | |
| 5 | Tetra-D-Lys  Tri-D-Arg | 1069.5027  1026.4717 | 1068.9922  1026.3213 | MS/MS: loss of 174 (terminal D-Arg) | |
| 7 | Tetra-D-Arg | 1097.5088 | 1097.4224 | MS/MS: loss of 174 (terminal D-Arg) | |
| 8 | TetraTriDapGly4 | 1779.7521 | 1779.3169 |  | |
| 9  9 | TriTriDap  TriTriDap-D-Lys | 1722.7306  1850.7892 | 1722.4832  1850.9132 | MS/MS: loss of 146 (terminal D-Lys) | |
|  | TriTriDap-D-Lys | 1850.7892 | 1850.8944 |  | |
| 10 | TriTriDap-D-Arg | 1878.8317 | 1878.7470 |  | |
|  | TetraTri | 1793.7677 | 1793.8154 |  | |
| 12 | TetraTri-D-Lys | 1921.8627 | 1922.0722 |  | |
| 13 | TetraTri-D-Lys | 1921.8627 | 1921.8816 |  | |
| 14 | TetraTri-D-Arg | 1949.8688 | 1949.9154 | MS/MS: loss of 174 (terminal D-Arg) | |
| 16 | TetraTetraTri/TetraTetraTriDap | 2717.1649 | 2717.0248 |  | |
| 18 | TriTriDap-D-Met | 1853.8580 | 1853.9370 | MS/MS: loss of 149 (terminal D-Met) | |
| 20 | TetraTri-D-Met | 1924.8952 | 1924.4164 | MS/MS: loss of 149 (terminal D-Met) | |
