# Supplementary material for: Septal Class A Penicillin-Binding Protein Activity and ld-Transpeptidases Mediate Selection of Colistin-Resistant Lipooligosaccharide-Deficient Acinetobacter baumannii
Source: mBio. 2021 Jan 5;12(1):e02185-20. doi: 10.1128/mBio.02185-20 (PMC8545086; doi:10.1128/mBio.02185-20)
Supplement: TABLE S3 [file mbio.02185-20-st003.docx]

| **Table S3:** Strains and plasmids used in this study | | |
| --- | --- | --- |
| **Strain/Plasmid** | **Description** | **Reference/Source** |
| **Strains** |  |  |
| *A. baumannii* 5075 | wild type | (1) |
| *A. baumannii* ACICU | wild type | (2) |
| *A. baumannii* ATCC 17978 | wild type | ATCC (3) |
| *A. baumannii* ATCC 19606 | wild type | ATCC (4) |
| *A. baumannii* AYE | wild type | ATCC (5) |
| *A. baumannii* SDF | wild type | ATCC (6) |
| *A. baumannii* 5075 | ABUW_LdtJ::Tn | (7) |
| *A. baumannii* 5075 | ABUW_LdtK::Tn | (7) |
| *A. baumannii* ATCC 17978 | Δ*mrcA* | (8) |
| *A. baumannii* ATCC 17978 | Δ*mrcA* / pPBP1A | (8) |
| *A. baumannii* ATCC 17978 | Δ*mrcA* / pPBP1A_E92Q_ | (8) |
| *A. baumannii* ATCC 17978 | Δ*mrcA* / pPBP1A_S459A_ | (8) |
| *A. baumannii* ATCC 17978 | Δ*mrcA* / pPBP1A OE | This study |
| *A. baumannii* ATCC 17978 | Δ*mrcB* | (8) |
| *A. baumannii* ATCC 17978 | Δ*mrcA* / pPBP1A-mCherry | This Study |
| *A. baumannii* ATCC 17978 | Δ*mrcA* / pPBP1A_E92Q_-mCherry | This Study |
| *A. baumannii* ATCC 17978 | Δ*mrcA* / pPBP1A_S459A_-mCherry | This Study |
| *A. baumannii* ATCC 17978 | Δ*ltdJ* | This Study |
| *A. baumannii* ATCC 17978 | Δ*ltdK* | This Study |
| *A. baumannii* ATCC 17978 | Δ*ltdJ* / pLtdJ | This Study |
| *A. baumannii* ATCC 17978 | Δ*ltdK* / pLtdK | This Study |
| *A. baumannii* ATCC 17978 | Δ*ltdJ* / pLtdJ_C390S_ | This Study |
| *A. baumannii* ATCC 17978 | Δl*tdK* / pLtdK_C138S_ | This Study |
| *A. baumannii* ATCC 19606 | LOS^-^ | (8) |
| *A. baumannii* ATCC 19606 | Δ*ltdJ* | This Study |
| *A. baumannii* ATCC 19606 | Δ*ltdK* | This Study |
| *A. baumannii* ATCC 19606 | Δ*ltdJ* / pLtdJ | This Study |
| *A. baumannii* ATCC 19606 | Δl*tdK* / pLtdK | This Study |
| **Plasmids** |  |  |
| pABBRKn | pABBR_ MCS with the *Kan^R^* gene from pKD4 replacing the *bla* gene, Kn^R^ | (8) |
| pMMBKn | pMMBKn_MCS with the *Kan^R^* gene from pKD4 replacing the *bla* gene, Kn^R^ | (8) |
| pABBRKn-mCherry | pABBRKn with the *mCherry* inserted into the KpnI and SacI sites, Kn^R^ | This Study |
| pPBP1A | pABBRKn with the *mrcA* gene and native promoter inserted into the XhoI and KpnI sites, Kn^R^ | (8) |
| pPBP1A OE | pMMBKn with the *mrcA* gene inserted into the XhoI and KpnI sites, under an IPTG inducible promoter, Kn^R^ | This Study |
| pPPBP1A_E92Q_ | pABBRKn with the *mrcA_E92Q_* and native promoter gene inserted into the XhoI and KpnI sites, Kn^R^ | (8) |
| pPBP1A_S459A_ | pABBRKn with the *mrcA_S459A_* gene and native promoter inserted into the XhoI and KpnI sites, Kn^R^ | (8) |
| pPBP1A-mCherry | pABBRKn-mCherry with the *mrcA_E92Q_* gene inserted into the XhoI and SacI sites, Kn^R^ | This Study |
| pPBP1A_E92Q_-mCherry | pABBRKn-mCherry with the *mrcA_E92Q_* gene inserted into the XhoI and SacI sites, Kn^R^ | This Study |
| pPBP1A_S459A_-mCherry | pABBRKn-mCherry with the *mrcA_S459A_* gene inserted into the XhoI and SacI sites, Kn^R^ | This Study |
| pAT03 | pMMB67EH with FLP recombinase, Amp^R^ | (9) |
| pAT04 | pMMB67EH with REC_Ab_ system, Tet^R^ | (9) |
| pKD4 | Kan^R^ | (10) |
| pLdtJ | pMMB67EH with the *ltdJ* gene and IPTG inducible promoter inserted into the KpnI and SalI sites, Kn^R^ | This Study |
| pLdtK | pMMB67EH with the *ltdK* gene and IPTG inducible promoter inserted into the KpnI and SalI sites, Kn^R^ | This Study |
| pLdtJ_C390S_ | pMMB67EH with the *ltdJ*_C390S_ gene and IPTG inducible promoter inserted into the KpnI and SalI sites, Kn^R^ | This Study |
| pLdtK_C138S_ | pMMB67EH with the *ltdK*_C138S_ gene and IPTG inducible promoter inserted into the KpnI and SalI sites, Kn^R^ | This Study |

**References**

1. Jacobs AC, Thompson MG, Black CC, Kessler JL, Clark LP, McQueary CN, Gancz HY, Corey BW, Moon JK, Si Y, Owen MT, Hallock JD, Kwak YI, Summers A, Li CZ, Rasko DA, Penwell WF, Honnold CL, Wise MC, Waterman PE, Lesho EP, Stewart RL, Actis LA, Palys TJ, Craft DW, Zurawski DV. 2014. AB5075, a Highly Virulent Isolate of *Acinetobacter baumannii,* as a Model Strain for the Evaluation of Pathogenesis and Antimicrobial Treatments. *mBio* 5:e01076-14.

2. Iacono M, Villa L, Fortini D, Bordoni R, Imperi F, Bonnal RJP, Sicheritz-Ponten T, De Bellis G, Visca P, Cassone A, Carattoli A. 2008. Whole-genome pyrosequencing of an epidemic multidrug-resistant *Acinetobacter baumannii* strain belonging to the European clone II group. *Antimicrob Agents Chemother* 52:2616–2625.

3. Baumann P, Doudoroff M, Stanier RY. 1968. A study of the Moraxella group. II. Oxidative-negative species (genus *Acinetobacter)*. *J Bacteriol* 95:1520–1541.

4. Bouvet PJM, Grimont PAD. 1986. Taxonomy of the Genus *Acinetobacter* with the Recognition of *Acinetobacter baumannii* sp. nov., *Acinetobacter haemolyticus* sp. nov., *Acinetobacter johnsonii* sp. nov., and *Acinetobacter junii* sp. nov. and Emended Descriptions of *Acinetobacter calcoaceticus* and *Acinetobacter lwoffii.* *International Journal of Systematic and Evolutionary Microbiology* 36:228–240.

5. Fournier P-E, Vallenet D, Barbe V, Audic S, Ogata H, Poirel L, Richet H, Robert C, Mangenot S, Abergel C, Nordmann P, Weissenbach J, Raoult D, Claverie J-M. 2006. Comparative genomics of multidrug resistance in *Acinetobacter baumannii*. *PLoS Genet* 2:e7.

6. Vallenet D, Nordmann P, Barbe V, Poirel L, Mangenot S, Bataille E, Dossat C, Gas S, Kreimeyer A, Lenoble P, Oztas S, Poulain J, Segurens B, Robert C, Abergel C, Claverie J-M, Raoult D, Médigue C, Weissenbach J, Cruveiller S. 2008. Comparative analysis of *Acinetobacters*: three genomes for three lifestyles. *PLoS ONE* 3:e1805.

7. Gallagher LA, Ramage E, Weiss EJ, Radey M, Hayden HS, Held KG, Huse HK, Zurawski DV, Brittnacher MJ, Manoil C. 2015. Resources for Genetic and Genomic Analysis of Emerging Pathogen *Acinetobacter baumannii*. *J Bacteriol* 197:2027–2035.

8. Boll JM, Crofts AA, Peters K, Cattoir V, Vollmer W, Davies BW, Trent MS. 2016. A penicillin-binding protein inhibits selection of colistin-resistant, lipooligosaccharide-deficient *Acinetobacter baumannii*. *Proc Natl Acad Sci USA* 113:E6228–E6237.

9. Tucker AT, Nowicki EM, Boll JM, Knauf GA, Burdis NC, Trent MS, Davies BW. 2014. Defining gene-phenotype relationships in *Acinetobacter baumannii* through one-step chromosomal gene inactivation. *mBio* 5:e01313-01314.

10. Datsenko KA, Wanner BL. 2000. One-step inactivation of chromosomal genes in *Escherichia coli* K-12 using PCR products. *Proc Natl Acad Sci USA* 97:6640–6645.
