# Supplementary material for: Septal Class A Penicillin-Binding Protein Activity and ld-Transpeptidases Mediate Selection of Colistin-Resistant Lipooligosaccharide-Deficient Acinetobacter baumannii
Source: mBio. 2021 Jan 5;12(1):e02185-20. doi: 10.1128/mBio.02185-20 (PMC8545086; doi:10.1128/mBio.02185-20)
Supplement: TABLE S4 [file mbio.02185-20-st004.docx]

| **Table S4:** Primers used in this study | | | |
| --- | --- | --- | --- |
| **Primer** | **Sequence 5' -> 3'** | **Description** | **Used to make** |
| PBP1A XhoI F | CGCTAACCTGAGCACACTATTGTCCTATTATTTTTTTATC | [CGC]-[XhoI]-[PBP1A native promoter; fwd] | pABBR PBP1A-mCherry |
| PBP1A EcoRI R | CGCGAATTCAATTTGATTAATCAAGTTTTCTAATTCATCTTTTTCACG | [CGC]-[EcoRI]-[PBP1A; rev] | pABBR PBP1A-mCherry |
| PBP1A KpnI F | CGCGGTACCATGAAAAAGCTATCCAGTTTGGGCTTCG | [CGC]-[KpnI]-[PBP1A; fwd] | pMMBKn PBP1A OE |
| PBP1A SalI F | CGCGTCGACTTAATCCTCAATTTGATTAATCAA | CGC]-[SalI]-[PBP1A; rev] | pMMBKn PBP1A OE |
| mCherry EcoRI F | CGCGAATTCGATAGCACTGAGAGCGGCTC | [CGC]-[EcoRI]-[mCherry; fwd] | pABBR PBP1A-mCherry |
| mCherry-Hix8x R | CGCGAGCTCCTAATGGTGATGGTGATGGTGATGGTGACTGGATCCGCTAGATCCCTGGG | [CGC]-[SacI]-[8xHis]-[mCherry; rev] | pABBR PBP1A-mCherry |
| A1S_2371 Mutagenesis Kan F | TTATATCCCTTCGCGTCTCAAATAAGCCAATATTAAATTCATAAGAATGAATGATTGGTGAGTTTATGGCCTAAAGGATCTGATTTTCCCTATTGCTTATATGAAAATTCTTAAGGTTGAATTACAGCGATTGTGTAGGCTGGAGCTGCTTCG | [Downstream A1S_2371]-[Kan] | *A. baumannii* ATCC 17978 Δ*ldtJ* |
| A1S_2371 Mutagenesis Kan R | TTAGTAAACCTAGGCTGGTTTTATTTTTATAATCAAAACAATAACTACATATTCCACGGGGCTATGCTAAAAAATTTAATAAAAAAGCCTGCATAAAGCAGGCTCTTTTAATTAAGAGGAATATCCTCCTTAGTTCCTATTCCG | [Upstream A1S_2371]-[Kan] | *A. baumannii* ATCC 17978 Δ*ldtJ* |
| A1S_2806 Mutagenesis Kan F | AAGCAGTGAAACTGCGCGTAGAGTAAAGAAGATTGATAATCTTTTAATTTCGGTGCAAGACAAGCTTTCCCATAATTTTTCATTAAAGCTCAAAATAGTTGAGTTGATATAAGAGAAGAAATTCGAGCGATTGTGTAGGCTGGAGCTGCTTCG | [Downstream A1S_2806]-[Kan] | *A. baumannii* ATCC 17978 Δ*ldtK* |
| A1S_2806 Mutagenesis Kan R | ATGCGAATGAATGATTAAAAATAAGTCATTATTAAGTAAAAAACAATTTTTTGTATTTTATTTAAGCACTTATTTTAATATTTTGATCATTCGGCTAATTAAACAATAATCTTTAAAATCCTATTATATCCTCCTTAGTTCCTATTCCG | [Upstream A1S_2806]-[Kan] | *A. baumannii* ATCC 17978 Δ*ldtK* |
| A1S_2371 KpnI F | CGCGGTACCATGTTTGTTCGCTCATTACTCGC | [CGC]-[KpnI]-[A1S_2371] | pMMB LdtJ complement |
| A1S_2371 SalI R | CGCGTCGACTTATTCTAAGAATTTAACAGTTA | [CGC]-[SalII]-[A1S_2371] | pMMB LdtJ complement |
| A1S_2806 KpnI F | CGCGGTATGTCCCAATTTAAACTTGAAGATG | [CGC]-[KpnI]-[ A1S_2806; fwd] | pMMB LdtK  complement |
| A1S_2806 SalI R | CGCGTCGACTTAGGTTAAAGATTGTTCAGACA | [CGC]-[SalI]- A1S_2806; rev] | pMMB LdtK  complement |
| A1S_2371 KpnI F | CGCGGTACCATGTTTGTTCGCTCATTACTCGC | [CGC]-[KpnI]-[A1S_2371] | pUC19 LtdJ |
| A1S_2371 SalI R | CGCGTCGACTTATTCTAAGAATTTAACAGTTA | [CGC]-[SalII]-[A1S_2371] | pUC19 LtdJ |
| A1S_2806 KpnI F | CGCGGTATGTCCCAATTTAAACTTGAAGATG | [CGC]-[KpnI]-[ A1S_2806; fwd] | pUC19 LtdK |
| A1S_2806 SalI R | CGCGTCGACTTAGGTTAAAGATTGTTCAGACA | [CGC]-[SalI]- A1S_2806; rev] | pUC19 LtdK |
| A1S_2371 C390S F | ATTTCTAAAACTGCATCACACGGTAGTATCCGTTTAACGAACTGGGATGCTAA | [A1S_2371]-[C390S]-[ A1S_2371; fwd] | pUC19 LtdJ_C390S_ |
| A1S_2371 C390S R | TTAGCATCCCAGTTCGTTAAACGGATACTACCGTGTGATGCAGTTTTAGAAAT | [A1S_2371]-[C390S]-[A1S_2371; rev] | pUC19 LtdJ_C390S_ |
| A1S_2806 C138S F | GATGGGGGTTCCAATGTCACATGGGAGTATTCGAATGCGTAATGAAGAAATCA | [A1S_2806]-[C138S]-[A1S_2806; fwd] | pUC19  LtdK_C138S_ |
| A1S_2806 C138S R | TGATTTCTTCATTACGCATTCGAATTGACCATGTGACATTGGAACCCCCATC | [A1S_2806]-[C138S]-[A1S_2806; rev] | pUC19  LtdK_C138S_ |
| A1S_2371 KpnI F | CGCGGTACCATGTTTGTTCGCTCATTACTCGC | [CGC]-[KpnI]-[A1S_2371] | pMMB LtdJ_C390S_ |
| A1S_2371 SalI R | CGCGTCGACTTATTCTAAGAATTTAACAGTTA | [CGC]-[SalII]-[A1S_2371] | pMMB LtdJ_C390S_ |
| A1S_2806 KpnI F | CGCGGTATGTCCCAATTTAAACTTGAAGATG | [CGC]-[KpnI]-[ A1S_2806; fwd] | pMMB LtdK_C138S_ |
| A1S_2806 SalI R | CGCGTCGACTTAGGTTAAAGATTGTTCAGACA | [CGC]-[SalI]- A1S_2806; rev] | pMMB LtdK_C138S_ |
